# Serum Metabolomics Study of Papillary Thyroid Carcinoma Based on HPLC-Q-TOF-MS/MS

**DOI:** 10.3389/fcell.2021.593510

**Published:** 2021-02-01

**Authors:** Yang Du, Peizhi Fan, Lianhong Zou, Yu Jiang, Xiaowen Gu, Jie Yu, Chaojie Zhang

**Affiliations:** ^1^Department of Breast and Thyroid Surgery, Hunan Provincial People's Hospital/The First Affiliated Hospital of Hunan Normal University, Changsha, China; ^2^Hunan Provincial Key Laboratory of Emergency and Critical Care Metabonomics, Institute of Emergency Medicine, Hunan Provincial People's Hospital/The First Affiliated Hospital of Hunan Normal University, Changsha, China

**Keywords:** thyroid papillary carcinoma, metabolite profile, serum samples, principal component analysis, orthogonal partial least square discriminant analysis

## Abstract

This study examined metabolite profile differences between serum samples of thyroid papillary carcinoma (PTC) patients and healthy controls, aiming to identify candidate biomarkers and pathogenesis pathways in this cancer type. Serum samples were collected from PTC patients (*n* = 80) and healthy controls (*n* = 80). Using principal component analysis (PCA), partial least squares discrimination analysis(PLS-DA), orthogonal partial least square discriminant analysis (OPLS-DA), *t*-tests, and the volcano plot, a model of abnormal metabolic pathways in PTC was constructed. PCA, PLS-DA, and OPLS-DA analysis revealed differences in serum metabolic profiles between the PTC and control group. OPLS-Loading plot analysis, combined with Variable importance in the projection (VIP)>1, Fold change (FC) > 1.5, and *p* < 0.05 were used to screen 64 candidate metabolites. Among them, 22 metabolites, including proline betaine, taurocholic acid, L-phenylalanine, retinyl beta-glucuronide, alpha-tocotrienol, and threonine acid were upregulated in the PTC group; meanwhile, L-tyrosine, L-tryptophan, 2-arachidonylglycerol, citric acid, and other 42 metabolites were downregulated in this group. There were eight abnormal metabolic pathways related to the differential metabolites, which may be involved in the pathophysiology of PTC. Six metabolites yielded an area under the receiver operating curve of >0.75, specifically, 3-hydroxy-cis-5-tetradecenoylcarnitine, aspartylphenylalanine, l-kynurenine, methylmalonic acid, phenylalanylphenylalanine, and l-glutamic acid. The Warburg effect was observed in PTC. The levels of 3-hydroxy-cis-5-tetradecenoylcarnitine, aspartylphenylalanine, l-kynurenine, methylmalonic acid, phenylalanine, and L-glutamic acid may help distinguish PTC patients from healthy controls. Aspartic acid metabolism, glutamic acid metabolism, urea cycle, and tricarboxylic acid cycle are involved in the mechanism of PTC.

## Introduction

Thyroid cancer is the most common type of endocrine tumor in clinical practice, accounting for 1.1% of all malignant tumors (Bray et al., 2018), while PTC is the most common type of thyroid cancer, accounting for ~90% of all cases. It is more common in women aged 30–45 years. PTC has good differentiation and low malignancy; however, it is prone to early lymph node metastasis (Ferlay et al., 2013). Therefore, early diagnosis and treatment are paramount to patient survival. Ultrasound-guided fine needle aspiration cytology (FNAC) is a commonly used auxiliary examination for the diagnosis of thyroid cancer. Although imaging tests have high sensitivity in the diagnosis of thyroid cancer, their specificity is poor (Remonti et al., 2015). FNAC is currently the most accurate and cost-effective method for assessing benign and malignant thyroid nodules; however, ~20–30% of cases cannot be confirmed as either benign or malignant by FNAC alone (Faquin, 2008; Fish, 2017). Because some papillary thyroid microcarcinomas have fewer abnormal cells, they may be missed or even misdiagnosed when only the FNAC method is performed (Kim et al., 2011). Therefore, several genetic tests have been proposed as useful in the diagnosis of thyroid cancer, including tests for *BRAF* and *NRAS* mutations, and *RET* translocation tests; in fact, *BRAF* mutations have been reported in 30–80% of PTC cases (Jin et al., 2006). However, samples from suspicious thyroid nodules that test negative for the *BRAF* gene mutation do not automatically exclude the possibility of PTC. In cases of inconclusive cytology findings, the detection of BRAF gene mutations can improve the rate of diagnosis of PTC (Johnson et al., 2014). Meanwhile, FNAC is an invasive examination, and the preoperative acceptance of patients is generally limited, but the clinical applicability of the latter is not very strong. Overall, this evidence indicates a need for a stable and reliable biomarker to assist in the diagnosis of thyroid cancer.

Metabolomics refers to a comprehensive analysis of the metabolome of biological systems under specific conditions. It is a type of high-throughput technology that plays an important role in systems biology research. The metabolome consists of thousands of complex molecular metabolites, whose relative molecular mass is <1 × 10^3^ (Barnes et al., 2016). High Performance Liquid Chromatography of Quadrupole Time of Flight Mass Spectrometry (HPLC-Q-TOF-MS/MS) combines liquid chromatography and mass spectrometry, thereby allowing to examine metabolites and perform stoichiometric analysis and improving the understanding of the molecular mechanisms of cancer development and associated biomarkers (Monteiro et al., 2013). The HPLC-Q-TOF-MS/MS platform allows for the separation and identification of complex mixtures, combining the compound separation capacity of the liquid chromatograph with the component identification ability of the mass spectrometer, resulting in high detection sensitivity and wide coverage of metabolite detection (Shepherd et al., 2011). Metabolomics is key to the understanding of the mechanisms of various cancers. For example, Yuan et al. (2018) used tandem mass spectrometry (UHPLC-MS/MS and FIA-MS/MS) to compare the types and levels of metabolites extracted from the plasma of patients with primary breast cancer with those of healthy controls, revealing that metabolites are mainly involved in amino acid metabolism and breast cancer cell growth pathways. Concurrently, Han et al. (2020) used UHPLC-MS/MS to show that retinol is a biomarker that distinguishes hepatocellular carcinoma (HCC) from adjacent tissues. The reported area under the curve associated with retinol (The area under curve, AUC = 0.991) suggests that it is important in HCC. Several previous studies have used metabolomics technology to distinguish PTC patients from healthy subjects; however, the studies had certain limitations. The advantages of the current study were as follows: (1). HPLC-Q-TOF-MS/MS is a novel method for identifying non-target metabolites, although it is less used in PTC research. (2). The sample size was sufficient. Using HPLC-Q-TOF-MS/MS to assess metabolic changes of PTC, we established a reliable statistical model that could distinguish and predict PTC patients and healthy controls. The main purpose of the study were as follows: (1). to identify metabolic markers that can distinguish PTC patients from healthy subjects using metabolomics; (2). to determine detailed metabolic changes and related metabolic pathways in PTC; and (3). to provide evidence for the diagnosis and treatment of PTC patients on the basis of science.

## Materials and Methods

### Patients and Study Design

This study complied with the guidelines of the Declaration of Helsinki and the Conference for Coordination of Clinical Practice and was approved by the Ethics Committee of Hunan Provincial People's Hospital. Each participant signed an informed consent form. The seventh edition of the American Joint Committee on Cancer Tumor-Lymph Node Metastasis staging system was used to determine PTC stage (Edge and Compton, 2010). This case-control study involved obtaining a serum sample from PTC patients undergoing total thyroidectomy at the study site between October 2018 and February 2020. Patient eligibility was confirmed based on pathological findings after thyroidectomy; only patients diagnosed with PTC were included; in contrast, patients with micro-PTC were excluded from this study. None of the patients had a history of another cancer, normal levels of thyroid hormones (T3 and T4), thyroid-stimulating hormone (TSH), no thyroid hormone medications before surgery, no other forms of cancer, immune blood system diseases, or metabolic disorders (metabolic syndrome, diabetes, and insulin resistance). Healthy controls visited the hospital to draw blood voluntarily for regular physical examinations. Healthy controls were recruited from among the individuals referred to the Saeed Pathobiology and Genetics Laboratory for routine examinations. Laboratory examination results confirmed normal levels of T3, T4, and TSH, and the absence of hypothyroidism, hyperthyroidism, nodular goiter, or autoimmune thyroid inflammation.

Each PTC patient provided ~5 ml of blood before the operation (patients were treatment-naïve at the time of sample collection); healthy controls provided blood samples after overnight fasting. Blood samples from both groups were stored for 2 h at 4°C and were subsequently centrifuged for 10 min at 4°C and 3,000 rpm. The centrifuged serum samples were extracted into 1.5 ml Eppendorf (Eppendorf Corporation, Germany)microtubes and stored at −80°C for later use.

### Serum Metabolite Extraction

Serum metabolites were extracted by adding 400 μl protein precipitant (MEthanol/ACN,v/v, 2:1) and 10 μl internal standard (L-2-chlorophenylalanine, 0.3 mg/ml, methanol preparation) to 100 μl of serum into 2 ml Eppendorf (Eppendorf Corporation, Germany)microtubes. The tube was vortexed for 30 s, ultrasonicated for 10 min (4°C water bath) and cooled at −20°C for 1 h. The tube was then centrifuged at 4°C at 13,000 rpm for 15 min to remove the precipitated protein.The supernatant of each sample was collected and stored in a refrigerator at −20°C.

Quality Control (QC) preparation: 10 μl was taken from each sample and added into 2 ml Eppendorf (Eppendorf Corporation, Germany)microtubes.Then vortex and divide into 200 μl for each tube.Quality control (QC) samples were pooled and pretreated using the same procedure to improve the data quality for metabolic profiling.

### HPLC-Q-TOF-MS/MS Metabolomics Analysis

HPLC-Q-TOF-MS/MS (Bruker Corporation, USA) was used as a metabolite separation and detection platform to study the metabolite differences between the PTC and control groups. The data were collected under the positive and negative ion modes of mass spectrometry. HPLC-Q-TOF-MS/MS conditions were the following: ACCLAINMTMRSLC120-C18 column (100 × 2.1 mm,2.2 μm) (Thermo fisher scientific, USA) at 40°C with 3 μl sample injection; mobile phase A was 0.1% (volume fraction) formic acid/water, and mobile phase B was 0.1% (volume fraction) acetonitrile/water (containing 0.1% formic acid). The gradient was set as follows: 2% B for 0–2 min, 50% B for 2–12 min, 90% B for 10–30 min, and 98% B for 30–60 min. The flow rate was maintained at 400 μl/min. The mass spectrometry conditions were as follows: Electrospray ion source was detected using positive and negative ion mode; high purity N2-assisted spray ionization and solvent removal was used; the flow rate was 1.2 l/min, the mass scanning range was 20–1,000 m/z, and the drying temperature was 200°C. In ESI positive mode, the Spray voltage (ISVF) is 4,500 V, and the capillary voltage is 100 V; in ESI negative ion mode, the Spray voltage (ISVF) is −4,500 V, and the capillary voltage is −100 V, fragmentor voltage 70 V.Quality control samples (QC samples) were analyzed five times at the beginning of the run and injected once after every 20 injections of the random sequenced samples.

### Raw HPLC-Q-TOF-MS/MS Data Processing

Metaboscape 3.0 (Bruker Corporation, USA) software was used to perform data cleaning, including peak extraction, noise reduction, standardization, and export, among others. Minimum Peak Length (3–5) spectrum; Recursive Feature Extraction/ Recursive Feature Extraction: Minimum Peak Length (Recursive) (5–7) spectrum. Minimum feature for extraction and presence of features in minimum of analyses will be selected according to the actual sample size. According to the 80% principle (Bijlsma et al., 2006), given 80 analyses, 64/80 analyses, retention time range [0, 30] min, and mass range [20, 1,000] M/Z. Ion Deconvolution/ Deconvolution inverse volume product EIC correlation ≥0.8. The ion fragments [such as [M+H]+, [M-H]–] were then recombined. Subsequently, the known false positive peaks, such as derivative chemical reagent peak, noise, and column loss peak, were removed from the data matrix; finally, the redundancy and peak combination procedures were performed. The data were uploaded to Annotate with Analyte List (HMDB database www.hmdb.ca), Annotate with Spectral Library [the standard product database created by Bruker [the most accurate]], and Annotate with Smartformula (online website database) database for matching and finally maintain the output A three-dimensional data matrix of time, mass response intensity, mass-to-nucleus ratio (M/Z), sample information, etc. This matrix was suitable for data analysis software such as SIMCAP, SPSS, and R language.

### Statistical Analysis

The metabolic profiles of serum samples were compared between the PTC and control groups, using multivariate and univariate analyses. Variable distribution was normalized using Log transformation and Pareto scaling for all pre-processed data. The Mann-Whitney-Wilcoxon test with false discovery rate correction was used to measure the significance of each metabolite. The SIMCAP14.1 software (Umetrics, Umea, Sweden) was used to PCA, PLS-DA, and OPLS-DA to determine the differences in metabolic profiles between the groups. The quality of the model is determined by the values of R2Y and Q2. R2Y represents the explanatory rate model, Q2 represents the forecast rate. Higher values of R2Y and Q2 usually indicate that the model is more reliable.Benjamini-Hochberg false discovery rate (FDR) procedure was employed for the multiple test adjustments. Adjusted *p* < 0.05 were considered statistically significant. Two hundred Permutation test was used to test model reliability. R^2^ and Q^2^ are obtained through permutations test, and its function is to verify whether the model is overfitting. When R^2^ > 0 and Q^2^ < 0, it means that the model is not overfitting and the model is reliable.VIP index represents the importance of each variable to model performance and describes the overall contribution of each variable to the model. Variables with a VIP of >1 have greater significance than do their counterparts. These variables were obtained from the PLS-DA model and adjusted *p* < 0.05.One-way analysis of variance and volcano plot were used to identify which metabolites annotated in the HPLC-Q-TOF-MS/MS dataset were significantly affected by the factor assessed in the experiment. MetaboAnalyst 4.0 (https://www.metaboanalyst.ca/) drew a heatmap, which was based on the estimates derived from the Spearman rank correlation and cluster analysis. The receiver operating characteristic (ROC) curve analysis was performed using the survival analysis module to evaluate the area under the curve (AUC) and to compare the diagnostic ability of significant metabolites between the tested groups.

### Metabolic Pathway Analysis

Use MetaboAnalyst 4.0 (https://www.metaboanalyst.ca/) on the difference of PTC patients serum and healthy subjects serum metabolites analysis of metabolic pathways, the purpose is to explain the biological correlation between PTC patients and healthy subjects. In this study, we referred to the Kyoto Encyclopedia of Gene and Genomes (KEGG, https://www.genome.jp/kegg) and the Human Metabolome Database (HMDB, https://hmdb.ca/) to elucidate any changes or interference patterns observed in the metabolic pathways in the study participants. KEGG is a knowledge base and is used for systematic analysis of metabolite function (Du et al., 2014). HMDB is a comprehensive database of metabolomics and metabolites biology (Wishart et al., 2013). MetaboAnalyst 4.0 combines enrichment and topology pathway analyses to identify relevant pathways. The module of pathway analysis was based on the KEGG database; the enrichment analysis was based on the Small Molecule Pathway Database (http://smpdb.ca/) (Jewison at al, 2014).

### Ethics Statement

The study was conducted in accordance with the Helsinki Declaration and was approved by the Ethics Committee of Human Provincial People's Hospital in Changsha, Hunan Province, China. Patients/participants provided their written informed consent to participate in the study.

## Results

### Clinical Characteristics of the Subjects

There were 80 PTC patients (18 men and 62 women; age range, 20–72 years), and 80 healthy controls (32 women and 48 men; age range, 30 and 67 years). The median age of the PTC and control groups was 41.63 ± 11.213 years and 43.44 ± 8.378 years, respectively; this difference was no statistically significant (*p* > 0.05) (Table 1).

**Table 1:** Clinical and pathological characteristic of the participants.

**Characteristic**	**PTC**	**Control (healthy subjects)**
Patient number	80	80
**Gender**		
Male	18	48
Female	62	32
**Age (Mean ± SD; year)**	41.63 ± 11.213	43.44 ± 8.378
**Clinical biochemistry tests (Mean ± SD)**	-	
TSH (μIU/ml)		1.79 ± 0.81
T4T (nmol/l)		110.51 ± 12.35
T3T (nmol/l)		2 ± 0.54
**Lymph node metastasis^a^**		-
Negative	26	
Positive	54	
**TNM stages^b^**		-
I	20	
II	52	
III	5	
IVA	3	

*PTC, papillary thyroid carcinoma*.

a *Includes N1a and/or N1b*.

b *American Joint Committee on Cancer (AJCC) Tumor-Node-Metastasis (TNM) staging system*.

### Serum Metabolomics Profiles in the PTC and Control Groups

After Metaboscape 3.0 pretreatment, a series of metabonomic data was obtained. In positive ion patterns, there were 384 identifiable peaks (Supplementary Figure 1), representing 384 detected metabolites. Supplementary Figure 1 shows the base peak chromatograms (BPC) of the PTC and control group serum samples. In negative ion patterns, there were 678 identifiable peaks (Supplementary Figure 2), representing 678 detected metabolites. Supplementary Figure 2 shows the BPC of the PTC and control group serum samples. There were significant between-peak differences in intensity, indicating that in the positive and negative ion mode, there were significant between-group differences in the metabolic profiles.

**Figure 1:**
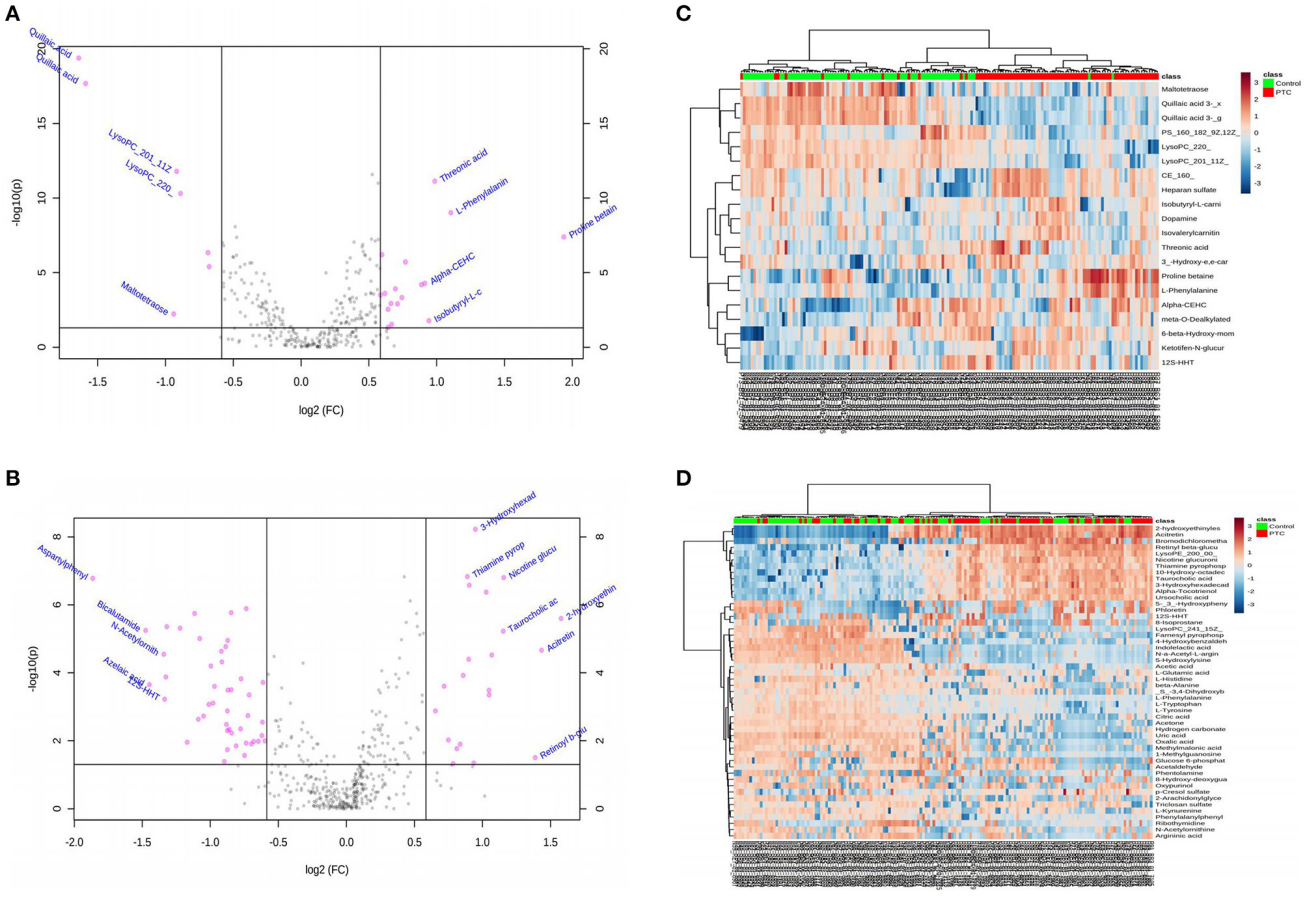
**(A,B)** Volcano diagrams showing the changes in PTC and the most important metabolites of healthy subjects. **(A)** Represents the volcano map in positive ion mode, and **(B)** represents the volcano map in negative ion mode. The red dots in the figure represent differential metabolites, and the black dots represent no significant difference. The red dot on the right side of the figure represents the upregulated metabolite, and the red dot on the left side represents the downregulated metabolite. x-axis corresponds to log_2_ (fold change) and y-axis corresponds to –log_10_ (*p-*value). **(C,D)** Heatmap visualization of metabolomics data with hierarchical clustering analysis (HCA). **(C)** Represents the heatmap in positive ion mode, and **(D)** represents the heatmap in negative ion mode. The red color represents the peak value that is relatively large; the blue color represents the peak value that is relatively small; and the gray color represents the metabolite peak value of zero. The more similar the color, the more similar the peak value. The panel on the right represents the different metabolites. The upper dendritic structure is clustered according to the degree of metabolite similarity across samples. The red line below the dendritic structure represents the PTC group, and the green line represents the control group. PTC, papillary thyroid carcinoma; Control, healthy subject.

Statistical tests commonly used to examine between-group differences in metabolite profiles include the *t*-test, FC analysis, and volcano plot. Univariate analysis can intuitively show the significance of different metabolites in two samples, and it is an essential statistical method in screening differential metabolites. A *p* < 0.05 was used to screen the different markers. Meanwhile, the volcano map (Figures 1A,B) was drawn based on the FC values and *t*-test findings (Supplementary Tables 1, 2). In the positive and negative ion mode, a total of 27 (Supplementary Table 1) and 73 different metabolites (Supplementary Table 2) were screened, respectively. Between-group differences in metabolites in the positive and negative ion modes were plotted as volcanic maps; red dots represent the differences in serum metabolites between the PTC and control groups. The volcanic map of metabolites given the positive and negative ion modes is shown in Figures 1A,B, respectively; there were 27 and 73 different metabolites, respectively. These findings indicated that amino acids, fatty acids and their derivatives, and nucleotides, among others, were the most important metabolites that differed between the groups. In particular, an increase in the levels of proline betaine, L-phenylalanine, threonic acid, isobutyryl-L-carnitine, and retinyl beta-glucuronide was observed in the PTC group, presenting candidate metabolic markers for differentiating PTC patients from healthy controls.

Hierarchical cluster analysis was used to cluster all metabolomic data with a *p* < 0.05 to examine the metabolites significantly changed between different groups. Within-group sample similarity was evaluated and presented as a heatmap obtained in positive and negative ion modes (Figures 1C,D, respectively). These data indicate specific patterns of differences in the metabolites between PTC and healthy controls.

### Screening of Differential Metabolites in Serum Samples Between the Two Groups

**Figure 2:**
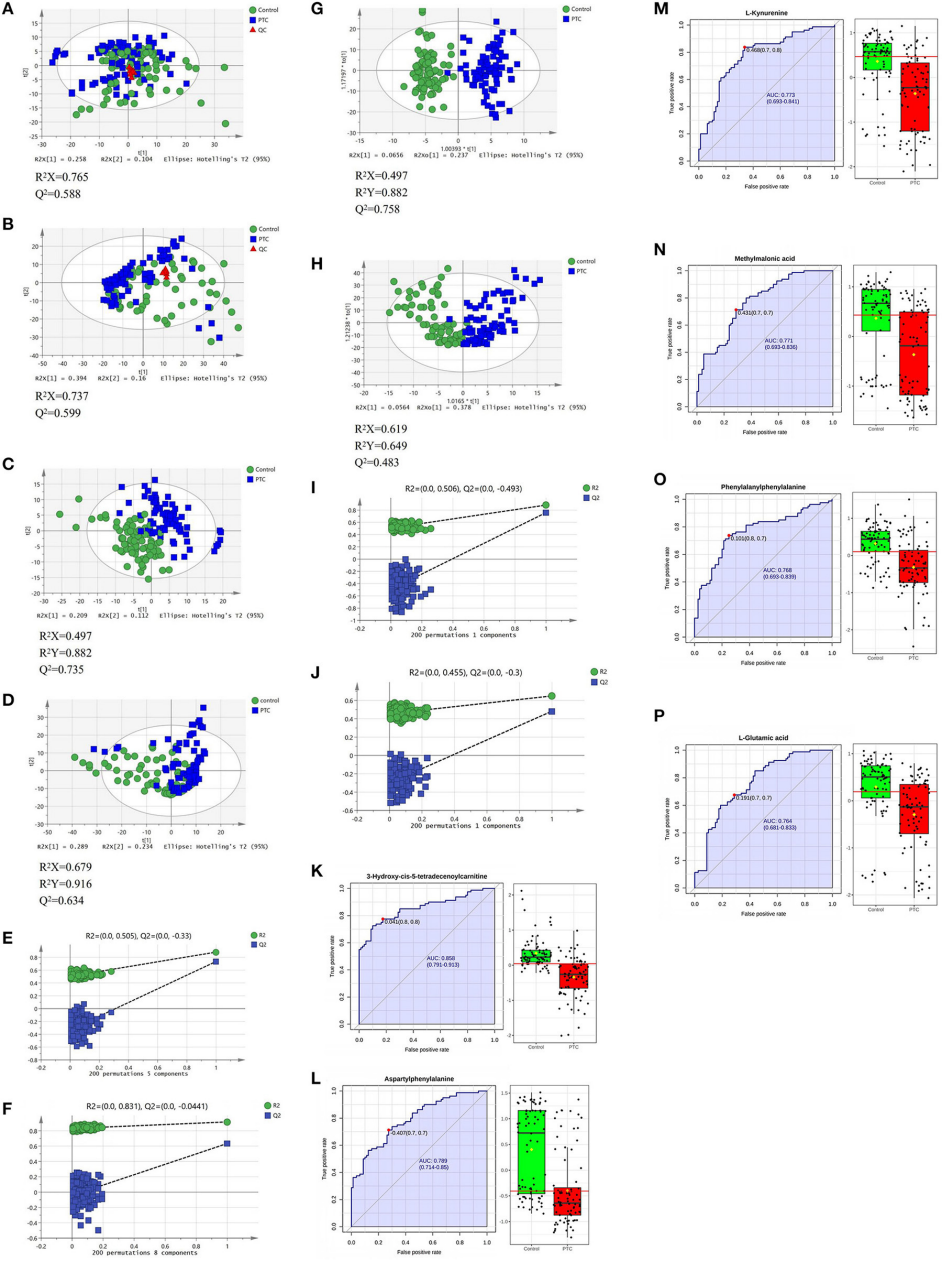
PCA **(A,B)**, PLS-DA **(C,D)**, and OPLS-DA **(G,H)** analysis score scatter plots illustrating that the metabolic profiles of PTC are distinct from those of healthy subjects. PLS-DA and OPLS-DA analysis score scatter plots for metabolic profiles of healthy subjects (green dots) and PTC patients (blue squares) showing clear discrimination between the two groups. **(E,F,I,J)** Permutation test was used to assess the reliability of the models. ROC curve analyses of the ability of six metabolites to predict PTC patients and healthy subject **(K–P)**. PCA, principal component analysis; PLS-DA, partial least squares discrimination analysis; OPLS-DA, orthogonal partial least squares-discriminant analysis; QC, Quality Control; PTC, papillary thyroid carcinoma; Control, healthy subject. ROC, receiver operating characteristic.

In the positive and negative ion mode, through the PCA model, we found that the clustering degree of the QC samples was good, indicating that the instrument was stable during this experiment. At the same time, we also found signs of separation between the PTC group and the Control group. The red triangle, green dot and blue square in the figure represent the QC group, the control group and the PTC group, respectively (Figures 2A,B).

Use the PLS-DA method to analyze the metabolite profile of the serum sample, as shown in Figure 1C: in the positive ion mode, the metabolomics data of serum samples were analyzed by PLS-DA, suggesting that there were significant differences between PTC and Control groups [R^2^X (cum) = 0.497, R^2^Y (cum) = 0.882, Q^2^ (cum) = 0.735). In the PLS-DA model, after 200 permutations tests, the *R*^2^ intercept of the substitution test in the positive ion mode was 0.505, and the intercept of Q^2^ was −0.33 (Figure 2E), suggesting model reliability, given no evidence of over-fitting;As shown in Figure 1D: in the negative ion mode, the metabolomics data of the PTC and Control groups also have significant differences between groups [R^2^X(cum) = 0.679, R^2^Y(cum) = 0.916, Q^2^(cum) = 0.634]. After 200 permutations tests, the R^2^ intercept of the substitution test in the positive ion mode was 0.831, and the intercept of Q^2^ was −0.0441 (Figure 2F), suggesting model reliability, given no evidence of over-fitting. These findings indicate that the PLS-DA model could be used to distinguish PTC patients from healthy controls; The parameters included in the model in both ion modes are shown in Supplementary Tables 3, 4.

To achieve the greatest separation of metabolites between the two sets of samples, OPLS-DA was performed (Figures 2G,H). In the positive and negative ion modes, there was a clear separation between the groups; concurrently, there were clear between-group differences in serum metabolic profiles. The samples from both groups tended to cluster in a concentrated manner, with a high degree of aggregation, without any obvious intragroup difference. In the OPLS-DA model, after 200 permutations tests, the R^2^ intercept of the substitution test in the positive ion mode was 0.506, and the intercept of Q^2^ was −0.493 (Figure 2I); the corresponding values in the negative ion mode were 0.455 and −0.3 (Figure 2J), suggesting model reliability, given no evidence of over-fitting. These findings indicate that the OPLS-DA model could be used to distinguish PTC patients from healthy controls; the model has a high discrimination and prediction rates (*P* < 0.05).

To distinguish the most important metabolites between the groups, FC, *p*-values, and VIP scores were used to screen for differential metabolites. In the positive and negative ion mode, the PCA-, PLS-DA-, and OPLS-DA-based models for distinguishing between the groups were constructed, and between-group metabolic differences were determined. Given VIP > 1.0, FC > 1.5, and *p* < 0.05, 64 metabolites were identified (Table 2). Among them, the levels of 22 metabolites showed a significant upward trend in the PTC group, including proline betaine, taurocholic acid, threonic acid, 3-hydroxyhexadecadienoylcarnitine, and dopamine. In contrast, the levels of 42 metabolites showed a significant downward trend in the PTC group, including L-tyrosine, 8-hydroxy-deoxyguanosine, 3-hydroxy-cis-5-tetradecenoylcarnitine, L-tryptophan, phenylalanylphenylalanine, argininic acid, beta-alanine, acetone, citric acid, and glucose 6-phosphate.

**Table 2:** Differential metabolites in the serum of papillary thyroid cancer patients and healthy subjects with positive and negative ion patterns.

**Name**	**Retention time (min)**	**Mass-to charge ratio**	**Formula**	**VIP**	**FC**	**log_**2**_(FC)**	**Adjusted *p* value**	**HMDB**	**ESI±**
Quillaic acid 3-[galactosyl-(1->2)-glucuronide]	14.01	413.2084	C42H64O16	2.11539	0.33217	−1.59	<0.001	HMDB0033404	+
Quillaic acid 3-[xylosyl-(1->3)-[galactosyl-(1->2)]-glucuronide]	14.06	479.248	C47H72O20	2.06349	0.32061	−1.6411	<0.001	HMDB0033406	+
Aspartylphenylalanine	1.33	279.09845	C13H16N2O5	1.84875	0.27591	−1.8577	<0.001	HMDB0000706	-
L-Histidine	1.33	154.06227	C6H9N3O2	1.8051	0.4009	−1.3187	<0.001	HMDB0000177	-
Pyridinoline	1.32	427.21866	C18H28N4O8	1.7665	0.40468	−1.3051	<0.001	HMDB0000851	-
5-hydroxylysine	11.39	161.10051	C6H14N2O3	1.66697	0.47622	−1.0703	<0.001	HMDB0000450	-
L-glutamic acid	1.06	146.04601	C5H9NO4	1.64934	0.51228	−0.96499	<0.001	HMDB0000148	-
Hydrogen carbonate	1.44	60.9925	CH2O3	1.62898	0.58709	−0.76835	<0.001	HMDB0000595	-
L-phenylalanine	7.39	166.08415	C9H11NO2	1.62402	2.0901	1.0636	<0.001	HMDB0000159	+
Trans-trans-Muconic acid	1.45	140.97822	C6H6O4	1.59533	0.53257	−0.90896	<0.001	HMDB0002349	-
Taurocholic acid	20.77	514.26972	C26H45NO7S	1.5798	2.2144	1.1469	<0.001	HMDB0000036	-
Disulfiram	1.5	294.904	C10H20N2S4	1.56751	0.61107	−0.7106	<0.001	HMDB0014960	-
Citric acid	1.43	190.95495	C6H8O7	1.5526	0.54732	−0.86954	0.0049857	HMDB0000094	-
Dimercaprol	1.45	122.96761	C3H8OS2	1.54495	0.52417	−0.9319	<0.001	HMDB0015677	-
Argininic acid	1.15	174.0885	C6H13N3O3	1.53498	0.5726	−0.80439	0.014188	HMDB0003148	-
Ursocholic acid	22.41	407.24778	C24H40O5	1.53168	1.8474	0.88546	<0.001	HMDB0000917	-
Methylmalonic acid	1.18	117.01943	C4H6O4	1.52339	0.42889	−1.2213	<0.001	HMDB0000202	-
Nicotine glucuronide	16.8	337.14296	C16H22N2O6	1.50428	2.2215	1.1515	<0.001	HMDB0001272	-
L-Kynurenine	1.32	207.08727	C10H12N2O3	1.48824	0.46308	−1.1107	<0.001	HMDB0000684	-
2-hydroxyethinylestradiol	11.97	311.16896	C20H24O3	1.47693	2.9752	1.573	<0.001	HMDB0061027	-
Oleoylcarnitine	22.06	424.33074	C25H47NO4	1.45757	2.0335	1.024	<0.001	HMDB0005065	-
Retinyl beta-glucuronide	22.05	461.30177	C26H38O7	1.45534	2.0624	1.0443	<0.001	HMDB0010340	-
N-a-Acetyl-L-arginine	11.97	215.10839	C8H16N4O3	1.4469	0.53088	−0.91355	<0.001	HMDB0004620	-
Cyanate	1.3	44.0489	CHNO	1.43683	1.8665	0.90033	<0.001	HMDB0002078	+
10-Hydroxy-octadec-12Z-enoate-9-beta-D-glucuronide	20.76	489.24827	C24H42O10	1.43271	2.0631	1.0448	<0.001	HMDB0060120	-
Biotin	11.97	243.10362	C10H16N2O3S	1.42499	0.60751	−0.71902	0.0018047	HMDB0000030	-
2-arachidonylglycerol	19.72	377.27343	C23H38O4	1.41697	0.55461	−0.85046	0.0046278	HMDB0004666	-
Beta-Alanine	1.18	88.04025	C3H7NO2	1.41179	0.55975	−0.83715	0.0059028	HMDB0000056	-
Indolelactic acid	1.35	204.06539	C11H11NO3	1.39434	0.48593	−1.0412	0.0018691	HMDB0000671	-
Acitretin	12.45	325.18468	C21H26O3	1.39063	2.6931	1.4293	<0.001	HMDB0014602	-
Hippuric acid	1.32	178.0511	C9H9NO3	1.3891	0.55416	−0.85163	<0.001	HMDB0000714	-
L-Tryptophan	1.33	203.08273	C11H12N2O2	1.38814	0.61765	−0.69513	0.0050668	HMDB0000929	-
Ribothymidine	1.14	257.07558	C10H14N2O6	1.372	0.49838	−1.0047	<0.001	HMDB0000884	-
3-Hydroxy-cis-5-tetradecenoylcarnitine	10.26	386.27558	C21H39NO5	1.37183	0.61797	−0.6944	0.012251	HMDB0013330	+
N-Acetylornithine	1.28	173.09307	C7H14N2O3	1.36098	0.39628	−1.3354	<0.001	HMDB0003357	-
Threonic acid	1.3	159.02767	C4H8O5	1.35864	1.9251	0.94496	<0.001	HMDB0000943	+
Oxalic acid	1.2	88.98794	C2H2O4	1.32474	0.50332	−0.99047	<0.001	HMDB0002329	-
Alpha-Tocotrienol	0.09	423.32682	C29H44O2	1.3208	1.9807	0.98599	<0.001	HMDB0006327	-
Acetone	1.42	56.99484	C3H6O	1.31729	0.54805	−0.86763	<0.001	HMDB0001659	-
4′-O-Methylepicatechin 7-O-glucuronide	7.17	238.07747	C23H26O11	1.31537	1.6712	0.74089	<0.001	HMDB0029183	-
Glucosylgalactosyl hydroxylysine	22.13	485.32744	C18H34N2O13	1.29406	1.6334	0.7079	0.0012046	HMDB0000585	-
L-Tyrosine	1.95	180.06682	C9H11NO3	1.29302	0.65647	−0.60719	<0.001	HMDB0000158	-
1-Methylguanosine	1.3	296.10035	C11H15N5O5	1.29055	0.60407	−0.72721	0.011676	HMDB0001563	-
Azelaic acid	1.34	187.09788	C9H16O4	1.29008	0.36788	−1.4427	<0.001	HMDB0000784	-
4-hydroxybenzaldehyde	1.34	121.02957	C7H6O2	1.2664	0.47227	−1.0823	0.0023175	HMDB0011718	-
(S)-3,4-Dihydroxybutyric acid	1.15	119.03508	C4H8O4	1.24485	0.6209	−0.68756	<0.001	HMDB0000337	-
Heparan sulfate	15.06	639.98222	C14H25NO21S3	1.22504	1.6421	0.71556	<0.001	HMDB0000693	+
4-glutathionyl cyclophosphamide	20.52	564.26422	C17H30Cl2N5O8PS	1.2227	0.6261	−0.67553	0.010508	HMDB0060387	-
Uric acid	1.29	167.02124	C5H4N4O3	1.19315	0.54884	−0.86554	<0.001	HMDB0000289	-
Thiamine pyrophosphate	17.05	424.07522	C12H19N4O7P2S	1.16992	1.8087	0.85496	<0.001	HMDB0001372	-
Phenylalanylphenylalanine	7.97	311.14035	C18H20N2O3	1.16405	0.60089	−0.73483	<0.001	HMDB0013302	-
Farnesyl pyrophosphate	1.14	381.10138	C15H28O7P2	1.16075	0.55865	−0.83999	<0.001	HMDB0000961	-
*p-*Cresol sulfate	1.33	187.00783	C7H8O4S	1.13682	0.44465	−1.1693	0.011129	HMDB0011635	-
3-hydroxyhexadecadienoylcarnitine	23.32	410.31521	C23H41NO5	1.11251	1.8723	0.90484	<0.001	HMDB0013335	-
Hesperetin 3′,7-O-diglucuronide	15.05	654.99189	C28H30O18	1.10556	1.6165	0.69291	<0.001	HMDB0041742	+
Glucose 6-phosphate	1.13	258.99652	C6H13O9P	1.08797	0.5463	−0.87223	0.0013207	HMDB0001401	-
Proline betaine	1.24	144.10164	C7H13NO2	1.08384	3.8372	1.9401	<0.001	HMDB0004827	+
Dopamine	1.29	154.08371	C8H11NO2	1.07989	0.66314	−0.59262	0.010018	HMDB0000073	+
3′-hydroxy-e,e-caroten-3-one	15.84	567.27116	C40H54O2	1.05843	1.5214	0.60537	<0.001	HMDB0002020	+
8-hydroxy-deoxyguanosine	1.24	282.08465	C10H13N5O5	1.05832	0.63158	−0.66296	0.0109	HMDB0003333	-
12(13)E*p-*9-KODE	11.93	309.1744	C18H30O4	1.04412	1.6818	0.75004	0.0092977	HMDB0013623	-
8-Isoprostane	19.38	279.44382	C20H40	1.03448	0.58342	−0.77739	0.00455	HMDB0004659	-
Maltotetraose	10.3	667.35056	C24H42O21	1.01631	0.52038	−0.94235	0.006033	HMDB0001296	+
Oxypurinol	1.32	151.02635	C5H4N4O2	1.00411	0.54446	−0.8771	0.003292	HMDB0000786	-

*The metabolites were listed in a decreasing order based on variable importance in the projection values (VIP). p value adjusted by false discovery rate method across all the metabolites within the comparison*.

Finally, ROC curve analysis was used to evaluate the diagnostic ability of the differential metabolites for PTC screening (Table 2). False positive rate and True positive rate are presented along the x-axis and y-axis, respectively. The AUC values of 6 metabolites in the PTC and control groups were of >0.75 (Figures 2K–P).

### Pathway Analysis

**Figure 3:**
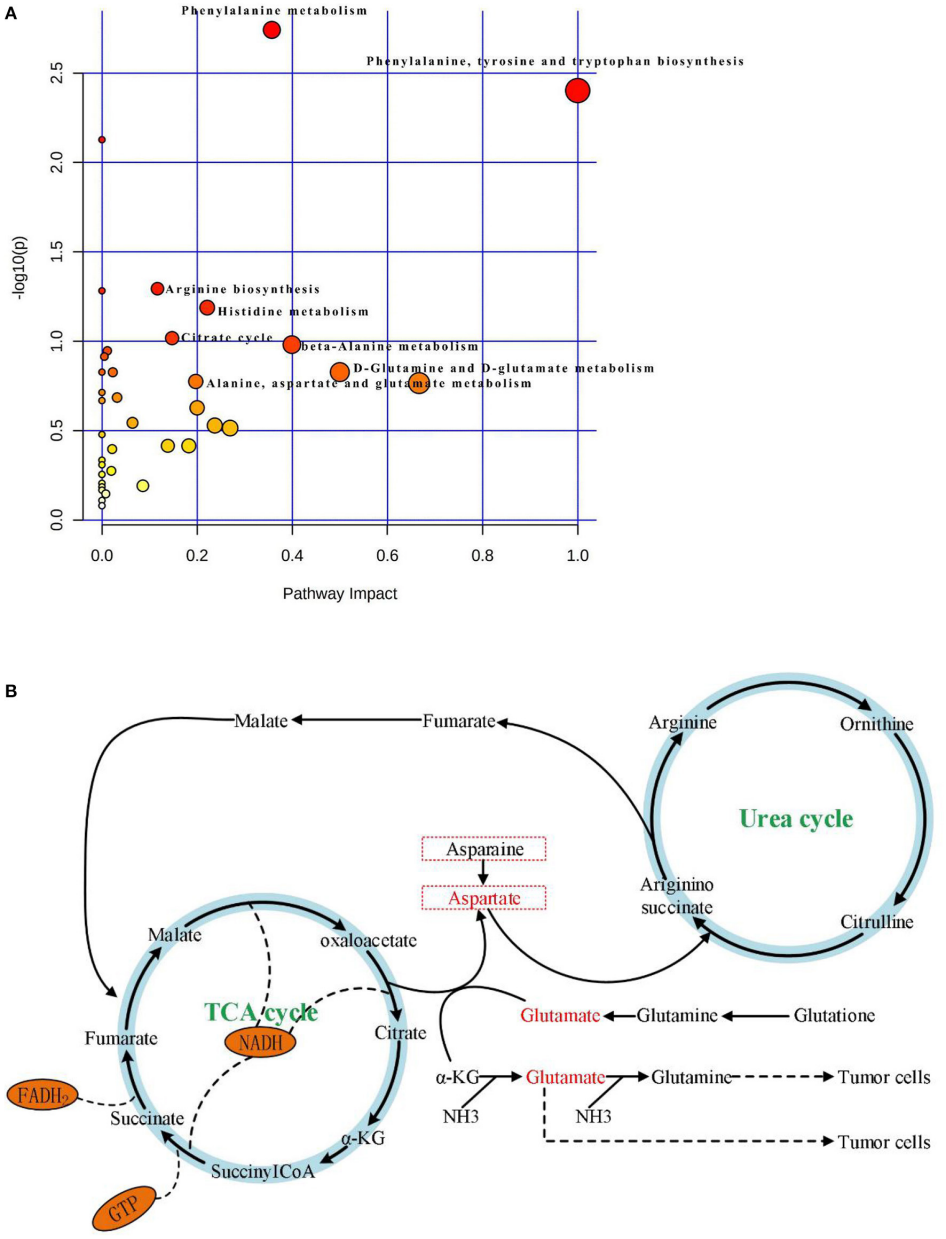
**(A)** Results of pathway analysis of metabolomics data. Pathway analysis based on “Kyoto Encyclopedia of Genes and Genomes” (KEGG).The color and size of each circle is based on *p-*values (yellow: higher *p-*values and red: lower *p-*values) and pathway impact values (the larger the circle the higher the impact score) calculated from the topological analysis, respectively. Pathways were considered significantly enriched if *p* < 0.05, impact >0.1 and number of metabolite hits in the pathway >1. PTC, papillary thyroid carcinoma. **(B)** The significantly enriched pathways involved in the pathogenesis of papillary thyroid carcinoma, including Aspartate metabolism, Glutamate metabolism, Urea cycle, and TCA cycle.

KEGG and HMDB (Table 2) were used to analyze 64 PTC-related metabolites, and the results were submitted to MetaboAnalyst to display the statistical analysis results of informatics analysis. The path analysis results are shown in Supplementary Table 5 and Figure 3A. The most influenced metabolic pathway was considered a pathway influence cut-off value >0.1 to filter for less important pathways. The following eight important metabolic pathways were identified: phenylalanine, tyrosine and tryptophan biosynthesis; D-glutamine and D-glutamate metabolism; beta-alanine metabolism; phenylalanine metabolism; histidine metabolism; alanine, aspartate, and glutamate metabolism; citrate cycle (TCA cycle); and arginine biosynthesis.

## Discussion

With the rapid development of analysis technology, metabolomics has been applied in many fields such as cancer disease research (Tayanloo-Beik et al., 2020). At the molecular level, metabolomics uses novel biomarkers to explore the mechanism underlying disease development (Wang et al., 2011). To the best of our knowledge, this is the first study to use HPLC-Q-TOF-MS/MS to analyze metabolic pathways of a large number of serum samples from PTC patients and healthy subjects. In this study, based on PCA, PLS-DA, OPLS-DA model results (Figures 2A–J), and single-factor analysis results (Figures 1A–D), we first identified key metabolites related to PTC (Table 2). Based on these key metabolites, six metabolic markers, namely 3-hydroxy-cis-5-tetradecenoylcarnitine, aspartylphenylalanine, l-kynurenine, methylmalonic acid, phenylalanylphenylalanine, and l-glutamic acid, that could distinguish PTC patients from healthy subjects were further identified (Figures 2K–P). At the same time, we discovered eight important metabolic pathways related to PTC (Figure 3A), which were involved in PTC development; however, the detailed metabolic changes remain unknown. We also found the association among aspartate metabolism, glutamate metabolism, urea cycle, and tricarboxylic acid cycle in the PTC metabolic pathway, thereby explaining the pathogenesis of PTC (Figure 3B).

Warburg (Hsu and Sabatini, 2008) reported that a large amount of energy is being produced by glycolysis during the growth of cancer cells, which is distinct from the energy metabolism observed in normal cells, where it involves oxidative phosphorylation. This finding suggests that the different growth patterns of cancer and normal cells may be due to the different energy production pathways involved. The rates of glucose uptake, aerobic glycolysis, and metabolism are increased in cancer cells due to cell proliferation (Zhao et al., 2010). The energy in healthy cells comes from the mitochondria that oxidize sugar molecules; in contrast, tumor cells mainly rely on glycolysis for energy, which does not require the participation of oxygen atoms or mitochondria (Gioia et al., 2019). According to Abooshahab et al. (2020), sucrose levels can separate PTC from benign thyroid tumors (AUC = 0.92). Sucrose is converted into glucose and fructose through the hydrolysis process; subsequently, glucose enters the aerobic glycolysis pathway, where it is converted into two molecules of pyruvate. In this study, the level of glucose 6-phosphate in the serum of PTC patients was lower than that in the serum of healthy subjects, which may indicate that PTC tumor cells obtain energy through enhanced glycolysis, which accelerates the conversion of glucose 6-phosphate into pyruvate molecules, required for the TCA cycle. The citric acid levels in the PTC group were lower than those in the control group, indicating that thyroid cancer cells consume a significant amount of citric acid during the TCA cycle. The present findings are consistent with the Warburg effect.

Glycerol phospholipids are the most abundant type of phospholipid in eukaryotic cell membranes. Together, phosphatidylcholine (PC) and phosphatidylethanolamine(PE) account for ~50% of the phospholipid components of cell membranes. In addition, glycerophospholipids are involved in protein recognition and signal transduction of cell membranes (Kuhajda, 2000). In malignant tumor tissues, as the rate of synthesis of glycerophospholipids is greater than that of their decomposition, choline substances are expressed at high levels (Treede et al., 2007). As a result of the destruction of membrane structures associated with the development of a malignant tumor, choline levels tend to increase (Wu et al., 2016). According to Wojakowska et al. (2017), PC and glycerophosphocholine are expressed at high levels in PTC tissues, and choline, the end product of metabolism, can generate new PC again. This process demonstrates that PTC also decomposes phospholipids while synthesizing phospholipids, with the goal of meeting the needs of cancer cell proliferation. In the present study, serum levels of 3-hydroxy-cis-5-tetradecenoylcarnitine in the PTC group were lower than in the control group. In addition, the associated AUC value was >0.865, indicating that 3-this metabolite may help distinguish PTC patients from healthy controls. Moreover, 3-hydroxy-cis-5-tetradecenoylcarnitine belongs to the carnitine group; carnitine and its short-chain derivatives are necessary for fatty acids to enter the mitochondria for oxidation. Cheng et al. (2018) has shown that 3-hydroxy-cis-5-tetradecenoylcarnitine can be used as an important biomarker for the diagnosis of bladder cancer (AUC and sensitivity values of 0.899 and 0.881, respectively). Nevertheless, the diagnostic validity of 3-hydroxy-cis-5-tetradecenoylcarnitine in PTC requires further studies to confirm. Arachidonylglycerol (2-AG) is an important endogenous cannabinoid, associated with abnormal metabolism in pancreatic ductal adenocarcinoma (Qiu et al., 2019), prostate cancer (Endsley et al., 2008), and HCC (Yang et al., 2019). Serum levels of 2-AG in the PTC group were lower than those in the control group. This finding might be accounted for by the fact that PTC tends to be characterized by a lower degree of malignancy than does HCC. Moreover, 2-AG has strong anti-proliferative and pro-apoptotic properties in PTC patients. A large amount of 2-AG is consumed in anti-proliferation and pro-apoptosis processes, resulting in the rate of 2-AG degradation higher than that of its synthesis. Further studies are required to verify these observations.

Cancer cells maintain cell growth and proliferation through different metabolic pathways. New cancer cells require a large number of biomolecular components, including proteins, nucleic acids, lipids, and important cofactors to maintain the redox state of cells. Amino acids are used by tumors as a source of nutrition during development; they can also be used as the main carbon source by new cancer cells (Kuhajda, 2000; Voeller et al., 2004). In the present study, the OPLS-DA model was used to screen amino acid-related differential metabolites, showing that the levels of tyrosine, tryptophan, arginine, alanine, glutamic acid, and histidine were lower in the PTC group than in the control group. Alanine is a glycogen amino acid that can be converted into an intermediate substrate in the tricarboxylic acid cycle, and then into glucose in the process of gluconeogenesis. Therefore, alanine can also be considered as an energy source for the rapid proliferation of PTC cells (Tian et al., 2015; Ryoo et al., 2016). In this study, serum levels of beta-alanine in the PTC group were lower than those in the control group. This finding might be accounted for by the fact that PTC cells use beta-alanine metabolism to convert large amounts of beta-alanine into raw materials for energy metabolism. Concurrently, this study examined amino acid metabolism in the context of energy metabolism. Aspartate and glutamate metabolism, and urea and TCA cycles emerged as important pathways in the development of PTC (Figure 3B). The metabolism of glutamate and aspartic acid is the most important participatory pathway in malignant thyroid tumors, linking the urea cycle with the TCA cycle. The urea cycle converts excess ammonia and aspartic acid into urea. Reduce the toxicity of its high ammonia content (Yekta et al., 2018). According to Nagamani and Erez (2016), in many malignant tumor tissues, the ASS1 enzyme is silenced in the urea cycle, which leads to the preferential synthesis of pyrimidine by aspartic acid to support cell proliferation, reducing the utilization of aspartic acid in pyrimidine synthesis, which limits the proliferation of cancer cells. Meanwhile, the silencing of the ASS1 enzyme in cancer cells supports their proliferation by activating carbamyl phosphate synthase-2, aspartate transcarbamylase, and the dihydrotransaminase complex, which promote pyrimidine synthesis (Rabinovich et al., 2015). This evidence suggests that silencing of the ASS1 enzyme is associated with poor prognosis in patients with malignant tumors. Ammonia plays an important role in the proliferation of PTC cells (Figure 3B). Glutamine provides ammonia and triggers autophagy in PTC cells. PTC cells generate glutamate through glutaminase and glutamate dehydrogenase, whereby glutamate further produces α-ketoglutarate, which provides sufficient energy for the survival of tumor cells. Glutamic acid and aspartic acid also undergo anaplerotic reactions, through which amino acids are oxidized and decomposed to generate intermediate metabolites of the TCA cycle, thereby supplying energy to tumor cells (Owen et al., 2002; Jones and Bianchi, 2015). A large number of studies has shown that in the proliferation of cancer cells, the metabolites of citric acid are transported out of the mitochondria, and used in lipid biosynthesis in the cytoplasm as a precursor of acetyl coenzyme-A to compensate for the continuous consumption of citric acid. Meanwhile, glutamine is the main anaplerotic precursor in cancer cells, compensating for the lack of citric acid, which is involved in energy generation (DeBerardinis et al., 2007). This study found that citric acid was significantly downregulated in PTC, as were glutamine and asparagine, reflecting the weakened replenishment response of glutamine in PTC. In addition, the levels of oxalic acid were lower in the PTC group than in the control group. The consumed oxalic acid was likely converted into an oxaloyl group, and the oxalic acid group was then converted into oxaloacetate. Oxalic acid is formed by combining an oxalyl group (after removing a hydroxyl group) and an acetic acid group. The acid cycle plays a catalyst-like role and determines the speed of the TCA cell cycle (Kuang et al., 2018). Oxaloacetic acid can also be transformed into non-essential amino acids such as asparate and asparagine, which are involved in nucleotide synthesis (Yang et al., 2017), suggesting that oxalic acid may be involved in PTC development.

In the present study, metabolomic and multivariate analyses were combined to distinguish PTC patients from healthy controls, aiming to determine the metabolic characteristics of PTC and improve the understanding of PTC development and associated prognosis. Future studies should include PTC tissue and lymph fluid analysis, and combine genomic and proteomic methods to yield further insights into PTC biomarkers and candidate treatment targets. Furthermore, future studies should involve accurate metabolomics analyses with a large number of specimens from PTC patients with lymph node metastasis, aiming to clarify the role of lymph node metastasis in PTC.

## Conclusions

We found that metabolomics based on HPLC-Q-TOF-MS/MS can clearly distinguish PTC patients from healthy subjects. Lower levels of 3-hydroxy-cis-5-tetradecenoylcarnitine, aspartylphenylalanine, l-kynurenine, methylmalonic acid, phenylalanylphenylalanine, and l-glutamic acid were observed in the serum of PTC patients than in the serum of healthy subjects. These six metabolic markers can theoretically be used in combination with current PTC diagnostic methods to guide the clinical diagnosis of PTC. However, we the following issues need to be considered: (1). For future clinical studies of PTC, it is necessary to further analyze the serum of PTC patients of phase III and IV to further confirm and summarize the results of this study; (2). Tissue and urine samples of PTC patients should be combined. The metabolomics research of lymphatic fluid can be used as a plan for future metabolomics research. This multicenter research aims to improve the accuracy of prediction.

## Data Availability Statement

The raw data supporting the conclusions of this article will be made available by the authors, without undue reservation.

## Ethics Statement

The studies involving human participants were reviewed and approved by Ethics Committee of Hunan Provincial People's Hospital. The patients/participants provided their written informed consent to participate in this study. Written informed consent was obtained from the individual(s) for the publication of any potentially identifiable images or data included in this article.

## Author Contributions

YD, LZ, and PF worked on the concept and design. XG, JY, and CZ have provided tools and patient specimens. YD, LZ, and YJ carried out experiments. YD and LZ analyzed and explained the results and edited the manuscript. YD organized the results and drafted the manuscript. LZ and PF approved the final version. All authors participated in the rigorous revision of manuscripts with important intellectual content.

## Conflict of Interest

The authors declare that the research was conducted in the absence of any commercial or financial relationships that could be construed as a potential conflict of interest.
