# Supplementary material for: Serum Metabolomics Study of Papillary Thyroid Carcinoma Based on HPLC-Q-TOF-MS/MS
Source: Front Cell Dev Biol. 2021 Feb 1;9:593510. doi: 10.3389/fcell.2021.593510 (PMC7882692; doi:10.3389/fcell.2021.593510)
Supplement: Supplementary file 1 [file Data_Sheet_1.docx]

**Supplementary Figure S1**


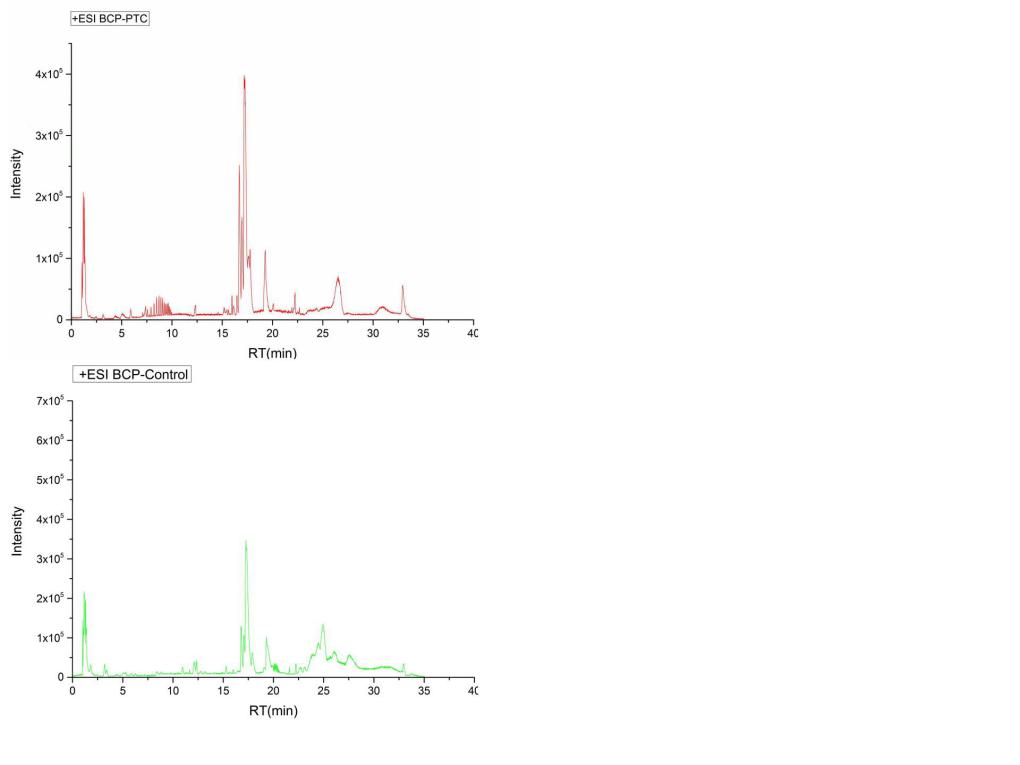


**Supplementary Figure S2**


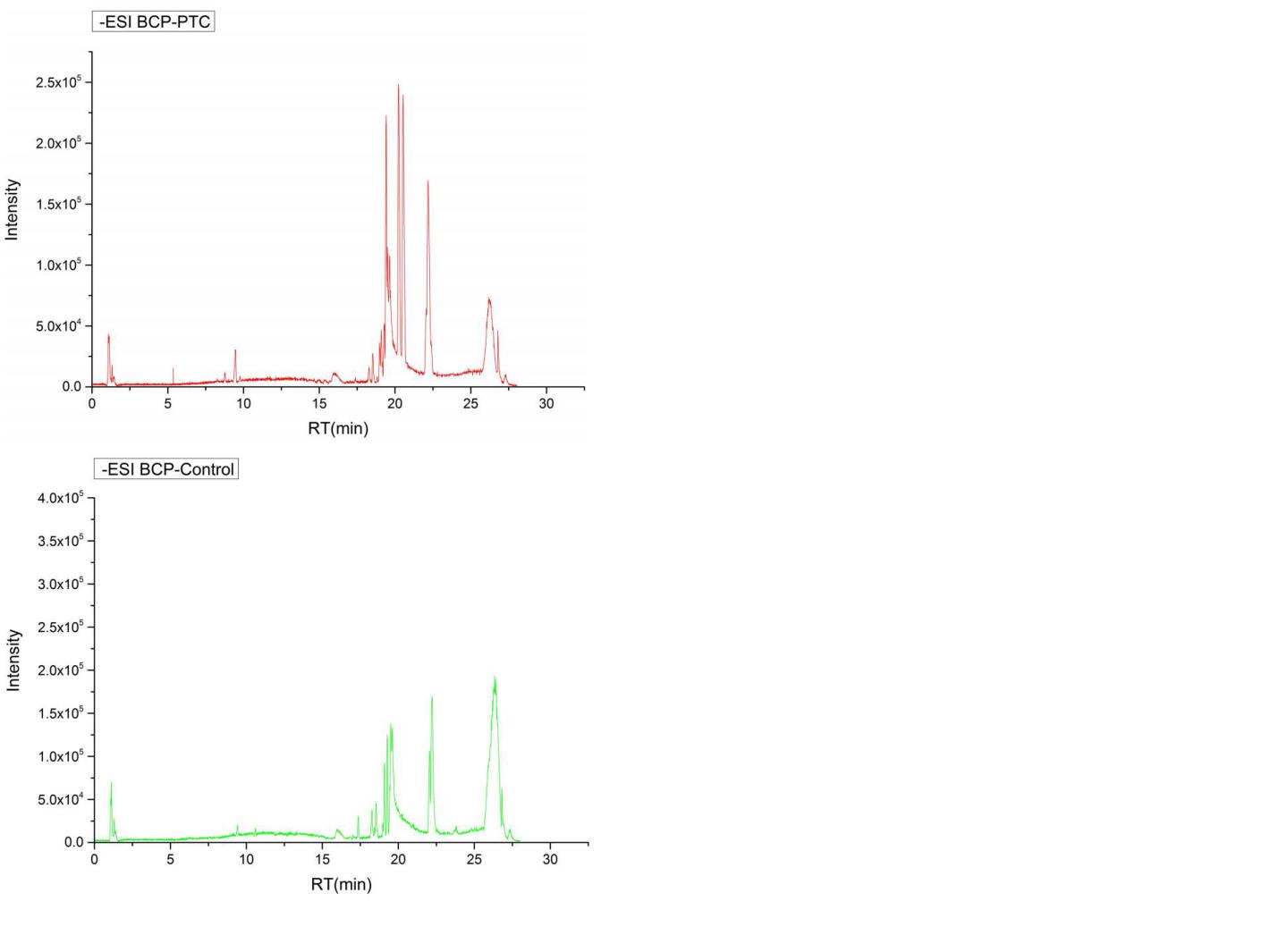


**Supplementary Table S1**

|  | FC | log2(FC) | p value |
| --- | --- | --- | --- |
| Quillaic acid 3-_xylosyl-_1-_3_-_galactosyl-_1-_2_-glucuronide_ | 0.32061 | -1.6411 | 3.80E-20 |
| Quillaic acid 3-_galactosyl-_1-_2_-glucuronide_ | 0.33217 | -1.59 | 2.28E-18 |
| LysoPC_201_11Z_ | 0.52836 | -0.9204 | 1.47E-12 |
| Threonic acid | 1.9807 | 0.98599 | 5.54E-12 |
| LysoPC_220_ | 0.53962 | -0.88997 | 4.92E-11 |
| L-Phenylalanine | 2.1536 | 1.1068 | 1.05E-09 |
| Proline betaine | 3.8372 | 1.9401 | 4.05E-08 |
| 3-Hydroxy-cis-5-tetradecenoylcarnitine | 0.6209 | -0.68756 | 3.96E-07 |
| Dopamine | 1.5214 | 0.60537 | 8.44E-07 |
| Isovalerylcarnitine | 1.7206 | 0.78293 | 2.49E-06 |
| PS_160_182_9Z,12Z_ | 0.62471 | -0.67874 | 3.63E-06 |
| 28-Glucosyloleanolic acid 3-_arabinosyl-_1-_2_-6-methylglucuronide_ | 0.65452 | -0.6115 | 3.82E-06 |
| Ketotifen-N-glucuronide | 1.5043 | 0.58912 | 5.81E-06 |
| Alpha-CEHC | 1.8944 | 0.92173 | 5.06E-05 |
| Cyanate | 1.8723 | 0.90484 | 6.51E-05 |
| 3_-Hydroxy-e,e-caroten-3-one | 1.6165 | 0.69291 | 0.000113 |
| Saprisartan | 1.6245 | 0.70003 | 0.000203 |
| meta-O-Dealkylated flecainide | 1.5457 | 0.62826 | 0.000243 |
| 6-beta-Hydroxy-mometasone furoate | 1.5044 | 0.58922 | 0.000308 |
| Heparan sulfate | 1.6712 | 0.74089 | 0.000456 |
| 12S-HHT | 1.5889 | 0.66806 | 0.001116 |
| Hesperetin 3_,7-O-diglucuronide | 1.6334 | 0.7079 | 0.001205 |
| Homovanillic acid | 1.5689 | 0.64971 | 0.002752 |
| Maltotetraose | 0.52038 | -0.94235 | 0.006033 |
| Isobutyryl-L-carnitine | 1.9544 | 0.96671 | 0.017252 |
| CE_160_ | 1.5859 | 0.66534 | 0.029222 |
| Adrenoyl ethanolamide | 1.578 | 0.65811 | 0.045927 |

**Supplementary Table S2**

|  | FC | log2(FC) | p value |
| --- | --- | --- | --- |
| Pyridinoline | 0.40468 | -1.3051 | 1.09E-09 |
| 3-Hydroxyhexadecadienoylcarnitine | 1.9251 | 0.94496 | 5.27E-09 |
| Thiamine pyrophosphate | 1.8474 | 0.88546 | 1.40E-07 |
| Nicotine glucuronide | 2.2215 | 1.1515 | 1.51E-07 |
| Aspartylphenylalanine | 0.27591 | -1.8577 | 1.64E-07 |
| Ursocholic acid | 1.8665 | 0.90033 | 2.46E-07 |
| Alpha-Tocotrienol | 2.0335 | 1.024 | 4.03E-07 |
| Phenylalanylphenylalanine | 0.60089 | -0.73483 | 1.24E-06 |
| Farnesyl pyrophosphate | 0.55865 | -0.83999 | 1.70E-06 |
| L-Kynurenine | 0.46308 | -1.1107 | 1.75E-06 |
| 2-hydroxyethinylestradiol | 2.9752 | 1.573 | 2.53E-06 |
| Methylmalonic acid | 0.42889 | -1.2213 | 4.83E-06 |
| Taurocholic acid | 2.2144 | 1.1469 | 5.84E-06 |
| 5-Hydroxylysine | 0.47622 | -1.0703 | 9.67E-06 |
| Acetone | 0.54805 | -0.86763 | 1.10E-05 |
| Acetic acid | 0.54229 | -0.88286 | 1.70E-05 |
| Acitretin | 2.6931 | 1.4293 | 2.17E-05 |
| N-a-Acetyl-L-arginine | 0.53088 | -0.91355 | 2.30E-05 |
| N-Acetylornithine | 0.39628 | -1.3354 | 2.80E-05 |
| 10-Hydroxy-octadec-12Z-enoate-9-beta-D-glucuronide | 2.0901 | 1.0636 | 2.96E-05 |
| LysoPE_200_00_ | 1.8611 | 0.89619 | 3.96E-05 |
| 6beta-hydroxybudesonide | 1.8961 | 0.92306 | 4.21E-05 |
| trans-trans-Muconic acid | 0.53257 | -0.90896 | 4.74E-05 |
| Oxalic acid | 0.50332 | -0.99047 | 6.24E-05 |
| Hippuric acid | 0.55416 | -0.85163 | 0.000111 |
| 12_13_Ep-9-KODE | 1.8087 | 0.85496 | 0.000118 |
| L-Histidine | 0.4009 | -1.3187 | 0.000132 |
| Hydrogen carbonate | 0.58709 | -0.76835 | 0.000149 |
| L-Phenylalanine | 0.65647 | -0.60719 | 0.000185 |
| Azelaic acid | 0.36788 | -1.4427 | 0.000223 |
| Glucosylgalactosyl hydroxylysine | 1.6421 | 0.71556 | 0.000245 |
| L-Glutamic acid | 0.51228 | -0.96499 | 0.000249 |
| Oxyphenonium | 1.7717 | 0.82515 | 0.000256 |
| 3_-Amino-3_-deoxythimidine glucuronide | 0.33256 | -1.5883 | 0.000267 |
| Citalopram N-oxide | 2.1224 | 1.0857 | 0.000308 |
| Oleoylcarnitine | 2.0624 | 1.0443 | 0.00032 |
| Uric acid | 0.54884 | -0.86554 | 0.000321 |
| Dimercaprol | 0.52417 | -0.9319 | 0.000402 |
| Triclosan sulfate | 0.61107 | -0.7106 | 0.000443 |
| Retinyl beta-glucuronide | 2.0631 | 1.0448 | 0.000447 |
| 12S-HHT | 0.3962 | -1.3357 | 0.000611 |
| LysoPC_241_15Z_ | 0.5091 | -0.97399 | 0.000783 |
| Ribothymidine | 0.49838 | -1.0047 | 0.000848 |
| Bromodichloromethane | 1.5747 | 0.65511 | 0.001312 |
| Glucose 6-phosphate | 0.5463 | -0.87223 | 0.001321 |
| Biotin | 0.60751 | -0.71902 | 0.001805 |
| Iloprost | 1.7238 | 0.78563 | 0.001857 |
| Indolelactic acid | 0.48593 | -1.0412 | 0.001869 |
| 4-Hydroxybenzaldehyde | 0.47227 | -1.0823 | 0.002318 |
| Oxypurinol | 0.54446 | -0.8771 | 0.003292 |
| 8-Isoprostane | 0.58342 | -0.77739 | 0.00455 |
| 2-Arachidonylglycerol | 0.55461 | -0.85046 | 0.004628 |
| Citric acid | 0.54732 | -0.86954 | 0.004986 |
| Disulfiram | 0.61765 | -0.69513 | 0.005067 |
| beta-Alanine | 0.55975 | -0.83715 | 0.005903 |
| Acetaldehyde | 0.65153 | -0.61809 | 0.006994 |
| 4_-O-methyl-_-_-epicatechin-7-O-beta-glucuronide | 1.6818 | 0.75004 | 0.009298 |
| L-Tyrosine | 0.66314 | -0.59262 | 0.010018 |
| 8-Hydroxy-deoxyguanosine | 0.64059 | -0.64252 | 0.010409 |
| _S_-3,4-Dihydroxybutyric acid | 0.6261 | -0.67553 | 0.010508 |
| 4-Glutathionyl cyclophosphamide | 0.63158 | -0.66296 | 0.0109 |
| p-Cresol sulfate | 0.44465 | -1.1693 | 0.011129 |
| 1-Methylguanosine | 0.60407 | -0.72721 | 0.011676 |
| L-Tryptophan | 0.61797 | -0.6944 | 0.012251 |
| LysoPC_224_7Z,10Z,13Z,16Z_ | 1.7802 | 0.83207 | 0.012512 |
| Argininic acid | 0.5726 | -0.80439 | 0.014188 |
| Trihydroxycoprostanoic acid | 1.7583 | 0.81422 | 0.016879 |
| 5-_3_-Hydroxyphenyl_-gamma-valerolactone-3_-O-glucuronide | 0.5464 | -0.87196 | 0.018257 |
| Phentolamine | 0.59706 | -0.74405 | 0.027038 |
| Retinoyl b-glucuronide | 2.6191 | 1.389 | 0.031072 |
| Phloretin | 0.53769 | -0.89514 | 0.040884 |
| LysoPC_201_11Z_ | 1.9156 | 0.93783 | 0.044884 |
| Decanoylcarnitine | 1.7219 | 0.78399 | 0.046818 |

**Supplementary Table S3**

| Var ID (Primary) | Var ID (Var. Sec. ID:1) | M2.VIP[1+4+0] | 2.44693 * M2.VIP[1]cvSE |
| --- | --- | --- | --- |
| Var_317 | Quillaic acid 3-_galactosyl-_1-_2_-glucuronide_ | 2.11539 | 0.507826 |
| Var_318 | Quillaic acid 3-_xylosyl-_1-_3_-_galactosyl-_1-_2_-glucuronide_ | 2.06349 | 0.51855 |
| Var_356 | Tetradecanedioic acid | 1.86112 | 0.487543 |
| Var_213 | LysoPC_22:0_ | 1.84983 | 0.302484 |
| Var_211 | LysoPC_20:1_11Z_ | 1.77026 | 0.373136 |
| Var_8 | 19-Hydroxyandrost-4-ene-3,17-dione | 1.71756 | 0.302939 |
| Var_250 | Neomycin | 1.59676 | 0.468472 |
| Var_26 | 3-Hydroxytetradecanedioic acid | 1.5894 | 0.227789 |
| Var_208 | LysoPC_18:1_11Z_ | 1.58756 | 0.255909 |
| Var_97 | Cidofovir | 1.58095 | 0.837002 |
| Var_117 | Dextrorphan O-glucuronide | 1.57034 | 0.358473 |
| Var_334 | Secobarbital | 1.51511 | 0.108715 |
| Var_142 | Flumethasone Pivalate | 1.50817 | 0.365788 |
| Var_101 | Cortexolone | 1.49691 | 0.327053 |
| Var_205 | Lubiprostone | 1.48815 | 0.312431 |
| Var_225 | Metabolite M6 | 1.48534 | 0.266386 |
| Var_210 | LysoPC_18:3_6Z,9Z,12Z_ | 1.4803 | 0.490038 |
| Var_57 | Amcinonide | 1.47157 | 0.244042 |
| Var_380 | Vinblastine | 1.46794 | 0.279938 |
| Var_124 | Digitoxin | 1.46428 | 0.24236 |
| Var_251 | Netilmicin | 1.45972 | 0.517758 |
| Var_54 | Almitrine | 1.45965 | 0.397888 |
| Var_28 | 4,5-Dihydro-drospirenone-3-sulfate | 1.45963 | 0.302468 |
| Var_282 | PC_22:5_4Z,7Z,10Z,13Z,16Z_P-18:1_11Z_ | 1.45541 | 0.240685 |
| Var_50 | Acitretin | 1.44638 | 0.202603 |
| Var_190 | Lapatinib | 1.43996 | 0.385099 |
| Var_90 | CE_14:0_ | 1.41463 | 0.282184 |
| Var_25 | 3-Hydroxytamoxifen _Droloxifene_ | 1.41456 | 0.319376 |
| Var_264 | O-Desmethylnaproxen | 1.40082 | 0.24722 |
| Var_309 | PS_18:3_9Z,12Z,15Z_18:1_9Z_ | 1.39867 | 0.308078 |
| Var_115 | Deslanoside | 1.39854 | 0.28019 |
| Var_283 | PC_o-16:1_9Z_18:2_9Z,12Z_ | 1.39182 | 0.284879 |
| Var_362 | Thromboxane B2 | 1.38157 | 0.467595 |
| Var_382 | Zafirlukast | 1.37409 | 0.344154 |
| Var_329 | Roxatidine acetate | 1.37237 | 0.47314 |
| Var_88 | Carmustine | 1.36155 | 0.512065 |
| Var_236 | Mivacurium | 1.35348 | 0.228276 |
| Var_136 | Eplerenone | 1.34828 | 0.454181 |
| Var_150 | gamma-Glutamylleucine | 1.33279 | 0.238357 |
| Var_374 | Tubocurarine | 1.32942 | 0.381923 |
| Var_360 | Threonic acid | 1.3208 | 0.595801 |
| Var_116 | Dexamethasone | 1.32 | 0.372076 |
| Var_18 | 2-trans,4-cis-Decadienoylcarnitine | 1.31791 | 0.392791 |
| Var_247 | N-Arachidonoyl glycine | 1.31764 | 0.280523 |
| Var_159 | Heparan sulfate | 1.31537 | 0.493906 |
| Var_123 | Dibucaine | 1.31206 | 0.32402 |
| Var_320 | Raffinose | 1.30418 | 0.39791 |
| Var_326 | Rifapentine | 1.30176 | 0.327447 |
| Var_151 | Gemifloxacin | 1.30138 | 0.389972 |
| Var_102 | Cortolone-3-glucuronide | 1.29521 | 0.250967 |
| Var_160 | Hesperetin 3_,7-O-diglucuronide | 1.29406 | 0.43029 |
| Var_75 | Bilirubin | 1.28862 | 0.272183 |
| Var_216 | LysoPC_24:1_15Z_ | 1.26816 | 0.317363 |
| Var_299 | Posaconazole | 1.26564 | 0.384825 |
| Var_375 | Uracil | 1.26561 | 0.471976 |
| Var_131 | Doxepin N-oxide glucuronide | 1.2592 | 0.403291 |
| Var_89 | Carvedilol | 1.25191 | 0.270193 |
| Var_306 | PS_16:0_18:2_9Z,12Z_ | 1.24952 | 0.299855 |
| Var_22 | 3-Hydroxy-cis-5-tetradecenoylcarnitine | 1.24485 | 0.512009 |
| Var_108 | Dabigatran etexilate | 1.24188 | 0.395929 |
| Var_14 | 28-Glucosylarjunolate 3-_rhamnosyl-_1-_3_-glucuronide_ | 1.24148 | 0.254237 |
| Var_125 | Dihydroergotamine | 1.2377 | 0.40478 |
| Var_263 | N-Trifluoroacetyladriamycin | 1.23523 | 0.433202 |
| Var_92 | CE_16:0_ | 1.23059 | 0.376337 |
| Var_121 | DG_18:2_9Z,12Z_22:4_7Z,10Z,13Z,16Z_0:0_ | 1.22313 | 0.402601 |
| Var_224 | Medicagenic acid 3-O-b-D-glucuronide | 1.22199 | 0.334652 |
| Var_1 | _2b,3b_-Dihydroxy-30-nor-12,20_29_-oleanadiene-28-glucopyranosyloxy-23-oic acid 3-glucuronide | 1.22134 | 0.552185 |
| Var_214 | LysoPC_22:2_13Z,16Z_ | 1.21959 | 0.381719 |
| Var_363 | Timolol | 1.21578 | 0.37824 |
| Var_260 | Norfloxacin | 1.21004 | 0.737102 |
| Var_239 | N2-gamma-Glutamylglutamine | 1.20081 | 0.78267 |
| Var_60 | Amphotericin B | 1.19132 | 0.370665 |
| Var_38 | 5-Acetylamino-6-formylamino-3-methyluracil | 1.19078 | 0.506545 |
| Var_13 | 25-O-Desacetyl rifabutin | 1.18359 | 0.628733 |
| Var_343 | Soyasapogenol B 3-O-b-D-glucuronide | 1.18185 | 0.417361 |
| Var_310 | PS_20:4_5Z,8Z,11Z,14Z_18:0_ | 1.17504 | 0.327883 |
| Var_373 | Tripelennamine | 1.17186 | 0.3439 |
| Var_189 | Lacosamide-glucuronide | 1.16339 | 0.306673 |
| Var_346 | Substance P | 1.16266 | 0.291618 |
| Var_70 | Atracurium | 1.15955 | 0.18191 |
| Var_96 | Chloroacetaldehyde | 1.15714 | 0.629717 |
| Var_20 | 31-Hydroxy rifabutin | 1.14416 | 0.245943 |
| Var_111 | Deoxycholic acid 3-glucuronide | 1.14394 | 0.441009 |
| Var_280 | PC_16:1_9Z_P-16:0_ | 1.14386 | 0.247998 |
| Var_167 | Hydroxy Ritonavir | 1.14235 | 0.558447 |
| Var_35 | 4R-Hydroxy solifenacin | 1.14056 | 0.279338 |
| Var_2 | 11-Oxo-androsterone glucuronide | 1.13583 | 0.362399 |
| Var_350 | Tamoxifen | 1.13374 | 0.333418 |
| Var_85 | Calcium | 1.12564 | 0.608369 |
| Var_84 | Butorphanol | 1.1231 | 0.366006 |
| Var_141 | Flavoxate | 1.12174 | 0.529048 |
| Var_106 | Cyanate | 1.11251 | 0.405313 |
| Var_53 | Aldosterone | 1.10814 | 0.396315 |
| Var_76 | Bimatoprost | 1.10808 | 0.371818 |
| Var_19 | 3_-Hydroxy-e,e-caroten-3-one | 1.10556 | 0.330926 |
| Var_233 | MG_18:2_9Z,12Z_0:0_0:0_ | 1.09715 | 0.618224 |
| Var_80 | Buspirone N-oxide | 1.09088 | 0.341866 |
| Var_41 | 6alpha,9alpha-Difluoroprednisolone-17-butyrate | 1.08543 | 0.379949 |
| Var_301 | Proline betaine | 1.08384 | 0.883754 |
| Var_158 | Guanidinosuccinic acid | 1.08177 | 0.53777 |
| Var_178 | Irinotecan | 1.08139 | 0.213606 |
| Var_314 | Pyridoxamine 5_-phosphate | 1.07888 | 0.411737 |
| Var_278 | PC_14:1_9Z_18:2_9Z,12Z_ | 1.0784 | 0.393599 |
| Var_196 | L-Glutamic acid | 1.07515 | 0.652259 |
| Var_34 | 4-Hydroxyvalsartan | 1.06713 | 0.695857 |
| Var_99 | cis-4-Octenedioic acid | 1.06407 | 0.273246 |
| Var_81 | Busulfan | 1.06038 | 0.340074 |
| Var_129 | Dopamine | 1.05843 | 0.419297 |
| Var_274 | Palonosetron | 1.04056 | 0.178999 |
| Var_209 | LysoPC_18:2_9Z,12Z_ | 1.03762 | 0.440136 |
| Var_344 | Spirapril | 1.03253 | 0.248407 |
| Var_308 | PS_18:1_9Z_22:6_4Z,7Z,10Z,13Z,16Z,19Z_ | 1.03095 | 0.323711 |
| Var_231 | Metronidazole | 1.03003 | 0.391226 |
| Var_201 | L-Phenylalanine | 1.02968 | 0.787531 |
| Var_286 | Pentosidine | 1.0268 | 0.380205 |
| Var_338 | SM_d18:0_18:0_ | 1.02301 | 0.191787 |
| Var_100 | Clofazimine glucuronide | 1.02291 | 0.235061 |
| Var_212 | LysoPC_20:2_11Z,14Z_ | 1.02067 | 0.347051 |
| Var_222 | Maltotetraose | 1.01631 | 0.823426 |
| Var_339 | SM_d18:1_18:1_11Z_ | 1.01603 | 0.166788 |
| Var_118 | Dextrothyroxine | 1.01256 | 0.23045 |
| Var_95 | Cer_d18:0_20:0_ | 1.01238 | 0.314672 |
| Var_323 | Rescinnamine | 1.01185 | 0.438265 |
| Var_62 | Amrinone | 1.00793 | 0.41656 |
| Var_276 | Paromomycin | 1.00469 | 0.218673 |
| Var_328 | Ritonavir | 1.00223 | 0.324318 |
| Var_277 | PC_14:0_P-18:0_ | 1.00039 | 0.264897 |

**Supplementary Table S4**

| Var ID (Primary) | Var ID (Name) | M3.VIP[1+3+0] | 2.44693 * M3.VIP[1]cvSE |
| --- | --- | --- | --- |
| Var_145 | Aspartylphenylalanine | 1.84875 | 0.309631 |
| Var_369 | L-Histidine | 1.8051 | 0.643432 |
| Var_102 | 6-Hydroxy-R-acenocoumarol | 1.69498 | 0.266142 |
| Var_94 | 5-Hydroxylysine | 1.66697 | 0.432195 |
| Var_379 | Lomustine | 1.66648 | 0.273711 |
| Var_308 | Fluorouracil | 1.66573 | 0.457832 |
| Var_368 | L-Glutamic acid | 1.64934 | 0.249081 |
| Var_339 | Hydrogen carbonate | 1.62898 | 0.17305 |
| Var_7 | 10-Hydroxy-octadec-12Z-enoate-9-beta-D-glucuronide | 1.62402 | 0.617932 |
| Var_485 | Oxychlordane | 1.61866 | 0.249036 |
| Var_450 | Niclosamide | 1.59818 | 0.215295 |
| Var_644 | trans-trans-Muconic acid | 1.59533 | 0.148344 |
| Var_409 | Mercaptopurine | 1.57994 | 0.477163 |
| Var_616 | Taurocholic acid | 1.5798 | 0.932355 |
| Var_650 | Triclosan sulfate | 1.56751 | 0.212974 |
| Var_204 | Citric acid | 1.5526 | 0.180736 |
| Var_142 | Argininic acid | 1.53498 | 0.42609 |
| Var_629 | Thiamine pyrophosphate | 1.53168 | 0.389045 |
| Var_405 | Magnesium salicylate | 1.52574 | 0.304068 |
| Var_419 | Methylmalonic acid | 1.52339 | 0.297909 |
| Var_58 | 3-Hydroxyhippuric acid | 1.50469 | 0.812322 |
| Var_451 | Nicotine glucuronide | 1.50428 | 0.435184 |
| Var_377 | L-Kynurenine | 1.48824 | 0.348738 |
| Var_45 | 2-hydroxyethinylestradiol | 1.47693 | 0.580687 |
| Var_170 | Caffeic acid 3-sulfate | 1.46746 | 0.252312 |
| Var_248 | D-Galactose | 1.45932 | 0.24995 |
| Var_129 | Alpha-Tocotrienol | 1.45757 | 0.392693 |
| Var_474 | Oleoylcarnitine | 1.45534 | 0.39763 |
| Var_654 | Trimethaphan | 1.45339 | 0.442823 |
| Var_435 | N-a-Acetyl-L-arginine | 1.4469 | 0.222579 |
| Var_670 | Ursocholic acid | 1.43683 | 0.865413 |
| Var_415 | Methazolamide | 1.4328 | 0.421414 |
| Var_570 | Retinyl beta-glucuronide | 1.43271 | 0.373355 |
| Var_131 | Alvimopan | 1.42647 | 0.124086 |
| Var_161 | Biotin | 1.42499 | 0.459138 |
| Var_29 | 1-beta-hydroxymedroxyprogesterone | 1.41721 | 0.411573 |
| Var_41 | 2-Arachidonylglycerol | 1.41697 | 0.261616 |
| Var_154 | beta-Alanine | 1.41179 | 0.398701 |
| Var_551 | Pyrophosphate | 1.4048 | 0.298054 |
| Var_505 | PE_P-16:0e_0:0_ | 1.397 | 0.297875 |
| Var_351 | Indolelactic acid | 1.39434 | 0.254309 |
| Var_117 | Acitretin | 1.39063 | 0.679532 |
| Var_640 | Tolazamide | 1.38685 | 1.05115 |
| Var_403 | LysoPE_20:0_0:0_ | 1.37974 | 0.380941 |
| Var_572 | Ribothymidine | 1.372 | 0.264714 |
| Var_387 | L-Tryptophan | 1.37183 | 0.178074 |
| Var_283 | Eicosadienoic acid | 1.3703 | 0.458008 |
| Var_177 | CE_17:1_ | 1.36363 | 0.416515 |
| Var_439 | N-Acetylornithine | 1.36098 | 0.678992 |
| Var_57 | 3-Hydroxyhexadecadienoylcarnitine | 1.35864 | 0.849564 |
| Var_396 | LysoPC_22:4_7Z,10Z,13Z,16Z_ | 1.3573 | 0.488132 |
| Var_399 | LysoPC_24:1_15Z_ | 1.35247 | 0.586842 |
| Var_615 | Taurine | 1.33235 | 0.244087 |
| Var_481 | Oxalic acid | 1.32474 | 0.129885 |
| Var_520 | Phenol | 1.31794 | 0.740083 |
| Var_115 | Acetone | 1.31729 | 0.126141 |
| Var_364 | L-Dopa | 1.30705 | 0.525479 |
| Var_176 | CD 1790 | 1.30484 | 0.790357 |
| Var_314 | Fumaric acid | 1.2956 | 0.467159 |
| Var_513 | Perfluorohexane sulfonic acid | 1.29431 | 0.435255 |
| Var_383 | L-Phenylalanine | 1.29302 | 0.242365 |
| Var_667 | Uridine | 1.29208 | 0.170021 |
| Var_582 | Serotonin | 1.29174 | 0.217449 |
| Var_30 | 1-Methylguanosine | 1.29055 | 0.289454 |
| Var_149 | Azelaic acid | 1.29008 | 0.543933 |
| Var_309 | Fluticasone propionate | 1.28056 | 0.389639 |
| Var_44 | 2-Hydroxyadipic acid | 1.27894 | 0.428542 |
| Var_223 | Deoxyuridine | 1.27338 | 0.641837 |
| Var_632 | Thioxanthine monophosphate | 1.27207 | 0.526685 |
| Var_340 | Hydroxychloroquine | 1.27042 | 0.322933 |
| Var_78 | 4-Hydroxybenzaldehyde | 1.2664 | 0.5455 |
| Var_475 | Olmesartan | 1.26466 | 0.69108 |
| Var_661 | Udenafil | 1.25333 | 0.583022 |
| Var_467 | Nornicotine | 1.25224 | 0.396146 |
| Var_325 | Glyceraldehyde | 1.24678 | 0.205021 |
| Var_181 | Cefuroxime | 1.24356 | 0.368914 |
| Var_482 | Oxamniquine | 1.24283 | 0.324949 |
| Var_118 | Adrenic acid | 1.24222 | 0.325981 |
| Var_135 | Amrinone | 1.24011 | 0.318116 |
| Var_10 | 11-Ketoetiocholanolone | 1.23727 | 0.632366 |
| Var_106 | 8,11,14-Eicosatrienoic acid | 1.23231 | 0.329926 |
| Var_59 | 3-Hydroxyquinine | 1.22807 | 0.346435 |
| Var_86 | 5-Acetylamino-6-formylamino-3-methyluracil | 1.22505 | 0.167221 |
| Var_323 | Glucosylgalactosyl hydroxylysine | 1.22504 | 0.550722 |
| Var_4 | _S_-3,4-Dihydroxybutyric acid | 1.2227 | 0.376005 |
| Var_457 | Nitrendipine | 1.22133 | 0.707726 |
| Var_665 | Ureidoisobutyric acid | 1.21994 | 0.197253 |
| Var_519 | Phenethylamine glucuronide | 1.21855 | 0.14638 |
| Var_529 | Phytanic acid | 1.21068 | 0.903942 |
| Var_68 | 3-Nitrotyrosine | 1.20758 | 0.244817 |
| Var_545 | Propenoylcarnitine | 1.19993 | 0.195907 |
| Var_666 | Uric acid | 1.19315 | 0.23419 |
| Var_427 | MG_18:2_9Z,12Z_0:0_0:0_ | 1.19008 | 0.511612 |
| Var_333 | Halofantrine | 1.18497 | 0.379851 |
| Var_1 | _-_-Epicatechin 3_-O-glucuronide | 1.1837 | 0.508552 |
| Var_201 | Cis-8,11,14,17-Eicosatetraenoic acid | 1.17097 | 0.3147 |
| Var_14 | 12_13_Ep-9-KODE | 1.16992 | 0.333029 |
| Var_12 | 11Z-Eicosenoic acid | 1.16716 | 0.604652 |
| Var_523 | Phenylalanylphenylalanine | 1.16405 | 0.363673 |
| Var_301 | Farnesyl pyrophosphate | 1.16075 | 0.2846 |
| Var_243 | DG_18:1_9Z_14:1_9Z_0:0_ | 1.15819 | 0.257668 |
| Var_550 | Pyrocatechol sulfate | 1.15731 | 0.571386 |
| Var_490 | Pantothenic acid | 1.15601 | 0.453464 |
| Var_282 | Echothiophate | 1.15452 | 0.607098 |
| Var_438 | N-Acetylneuraminic acid | 1.1511 | 0.311052 |
| Var_580 | Saccharopine | 1.14996 | 0.681754 |
| Var_465 | Norfluoxetine | 1.14513 | 0.37284 |
| Var_360 | L-2-Hydroxyglutaric acid | 1.13908 | 0.197503 |
| Var_503 | p-Cresol sulfate | 1.13682 | 0.544196 |
| Var_639 | Tizanidine | 1.13361 | 0.585372 |
| Var_358 | Ketorolac | 1.12863 | 0.123804 |
| Var_540 | Polystyrene sulfonate | 1.12637 | 0.368581 |
| Var_390 | LysoPC_15:0_ | 1.12612 | 0.393856 |
| Var_209 | Conivaptan | 1.12503 | 0.304451 |
| Var_354 | Isodesmosine | 1.12334 | 0.368527 |
| Var_111 | Acetaldehyde | 1.11915 | 0.206067 |
| Var_269 | Docosatrienoic acid | 1.11749 | 0.457721 |
| Var_398 | LysoPC_22:6_4Z,7Z,10Z,13Z,16Z,19Z_ | 1.11341 | 0.744992 |
| Var_366 | Levamisole | 1.10985 | 0.314668 |
| Var_246 | DG_18:2_9Z,12Z_22:4_7Z,10Z,13Z,16Z_0:0_ | 1.10649 | 0.175355 |
| Var_121 | Alfuzosin | 1.09996 | 0.376922 |
| Var_552 | Pyruvic acid | 1.09953 | 0.386984 |
| Var_483 | Oxoadipic acid | 1.09139 | 0.200762 |
| Var_241 | DG_18:1_11Z_20:5_5Z,8Z,11Z,14Z,17Z_0:0_ | 1.09101 | 0.383667 |
| Var_5 | 1,3,7-Trimethyluric acid | 1.0895 | 0.072139 |
| Var_21 | 14R,15S-EpETrE | 1.08852 | 0.616649 |
| Var_322 | Glucose 6-phosphate | 1.08797 | 0.413821 |
| Var_316 | Gamma-Butyrolactone | 1.08277 | 0.243028 |
| Var_225 | Desloratadine | 1.08232 | 0.586126 |
| Var_222 | Dehydrogenated ticlopidine | 1.0808 | 0.180827 |
| Var_388 | L-Tyrosine | 1.07989 | 0.057899 |
| Var_9 | 10Z-Nonadecenoic acid | 1.07615 | 0.342359 |
| Var_421 | Methylsuccinic acid | 1.07588 | 0.385547 |
| Var_50 | 3,4-Dihydroxyphenylacetone | 1.07524 | 0.500825 |
| Var_326 | Glyceric acid | 1.07516 | 0.17511 |
| Var_63 | 3-Hydroxyvaleric acid | 1.07439 | 0.367383 |
| Var_324 | Glutathione | 1.07112 | 0.369866 |
| Var_416 | Methoxsalen | 1.07095 | 0.14578 |
| Var_413 | Methacycline | 1.07005 | 0.441982 |
| Var_237 | DG_16:0_22:5_7Z,10Z,13Z,16Z,19Z_0:0_ | 1.06648 | 0.664182 |
| Var_385 | L-Proline | 1.0634 | 0.254205 |
| Var_642 | trans-Aconitic acid | 1.0598 | 0.495689 |
| Var_233 | DG_14:1_9Z_20:3_8Z,11Z,14Z_0:0_ | 1.05894 | 0.296485 |
| Var_122 | Allantoin | 1.05867 | 0.225675 |
| Var_300 | Ezetimibe | 1.05797 | 0.76234 |
| Var_546 | Prostaglandin F1a | 1.0492 | 0.487207 |
| Var_277 | Drospirenone | 1.04851 | 0.117688 |
| Var_526 | Phosphoenolpyruvic acid | 1.04737 | 0.456685 |
| Var_575 | Risedronate | 1.04713 | 0.339813 |
| Var_72 | 4_-O-methyl-_-_-epicatechin-7-O-beta-glucuronide | 1.04412 | 0.602059 |
| Var_23 | 15-Methylpalmitate | 1.04394 | 0.107616 |
| Var_76 | 4-Hydroxy-5-_3_,4_-dihydroxyphenyl_-valeric acid-O-glucuronide | 1.03991 | 0.566385 |
| Var_477 | O-Phosphoethanolamine | 1.03911 | 0.26315 |
| Var_242 | DG_18:1_11Z_22:6_4Z,7Z,10Z,13Z,16Z,19Z_0:0_ | 1.03847 | 0.50907 |
| Var_27 | 18-Hydroxycorticosterone | 1.03799 | 0.434824 |
| Var_539 | Plerixafor | 1.03666 | 0.41501 |
| Var_55 | 3-Hydroxycapric acid | 1.03635 | 0.382128 |
| Var_108 | 8-Isoprostane | 1.03448 | 0.541577 |
| Var_607 | Sulfate | 1.03284 | 0.16705 |
| Var_584 | Sertraline | 1.03201 | 0.351487 |
| Var_389 | Lubiprostone | 1.02895 | 0.570885 |
| Var_218 | D-Aspartic acid | 1.02556 | 0.862847 |
| Var_404 | LysoPE_22:6_4Z,7Z,10Z,13Z,16Z,19Z_0:0_ | 1.02471 | 0.413116 |
| Var_274 | Doxazosin | 1.02007 | 0.28581 |
| Var_378 | L-Methionine | 1.01769 | 0.118547 |
| Var_107 | 8-Hydroxy-deoxyguanosine | 1.01423 | 0.410408 |
| Var_212 | Coumesterol | 1.01356 | 0.482493 |
| Var_630 | Thioridazine | 1.01088 | 0.565467 |
| Var_641 | Torasemide | 1.00986 | 0.492961 |
| Var_275 | Doxycycline | 1.00847 | 0.496862 |
| Var_125 | Allysine | 1.0063 | 0.431859 |
| Var_19 | 13-HOTE | 1.00457 | 0.801115 |
| Var_487 | Oxypurinol | 1.00411 | 0.316333 |
| Var_384 | L-Pipecolic acid | 1.0014 | 0.42166 |
| Var_184 | Ceramide _d18:1_18:0_ | 1.00045 | 0.603124 |
| Var_599 | Stearic acid | 0.998615 | 0.239693 |
| Var_133 | Amidosulfonic acid | 0.998286 | 0.222978 |
| Var_392 | LysoPC_20:1_11Z_ | 0.997012 | 0.629503 |
| Var_169 | Butethal | 0.995268 | 0.173052 |
| Var_612 | Talbutal | 0.992095 | 0.409341 |
| Var_155 | Betaine | 0.99193 | 0.274597 |
| Var_549 | Pyrocatechol | 0.988198 | 0.439423 |
| Var_213 | Creatinine | 0.986935 | 0.349199 |
| Var_312 | Formic acid | 0.986691 | 0.192006 |
| Var_279 | D-Urobilinogen | 0.982511 | 0.24529 |
| Var_577 | Rivastigmine | 0.982149 | 0.360463 |
| Var_173 | Carbimazole | 0.978627 | 0.183008 |
| Var_375 | Lithocholic acid | 0.978062 | 0.386773 |
| Var_93 | 5-Hydroxyindoleacetic acid | 0.977862 | 0.553569 |
| Var_426 | MG_0:0_18:1_9Z_0:0_ | 0.976819 | 0.645791 |
| Var_215 | Cystathionine ketimine | 0.97484 | 0.255338 |
| Var_556 | Quetiapine | 0.973613 | 0.696245 |
| Var_341 | Hydroxyisocaproic acid | 0.97289 | 0.257888 |
| Var_217 | Dalfopristin | 0.971925 | 0.370474 |
| Var_16 | 12S-HHT | 0.97091 | 0.731851 |
| Var_407 | Malonic acid | 0.967958 | 0.101733 |
| Var_647 | Triamcinolone | 0.967635 | 0.30201 |
| Var_13 | 12,13-DHOME | 0.966825 | 0.25525 |
| Var_507 | Penicillin V | 0.964304 | 0.67616 |
| Var_664 | Uracil mustard | 0.963263 | 0.165728 |
| Var_168 | Buprenorphine glucuronide | 0.961835 | 0.347412 |
| Var_456 | Nitrazepam | 0.958117 | 0.436285 |
| Var_20 | 14-HDoHE | 0.956357 | 0.392396 |
| Var_537 | Pipotiazine | 0.953956 | 0.206702 |
| Var_261 | Dihydroergotamine | 0.953046 | 0.491131 |
| Var_137 | Androsterone sulfate | 0.951589 | 0.615638 |
| Var_114 | Acetoacetic acid | 0.951239 | 0.34113 |
| Var_28 | 19-Noretiocholanolone | 0.950827 | 0.567324 |
| Var_156 | Betaxolol | 0.950022 | 0.232275 |
| Var_318 | Gamma-Linolenic acid | 0.948511 | 0.674837 |
| Var_522 | Phentolamine | 0.947196 | 0.42912 |
| Var_164 | Bretylium | 0.946892 | 0.123778 |
| Var_292 | Erythrityl Tetranitrate | 0.942686 | 0.371814 |
| Var_40 | 25-Hydroxyvitamin D2 | 0.942294 | 0.239364 |
| Var_512 | Perfluorodecanoic acid | 0.941626 | 0.137141 |
| Var_88 | 5a-Pregnane-3,20-dione | 0.940843 | 0.256024 |
| Var_357 | Isovalerylglycine | 0.94068 | 0.366787 |
| Var_622 | Tetrahydrocortisone | 0.937112 | 0.437548 |
| Var_66 | 3-Methyl-2-oxovaleric acid | 0.933389 | 0.309751 |
| Var_56 | 3-Hydroxy-cis-5-tetradecenoylcarnitine | 0.93281 | 0.506694 |
| Var_98 | 5-Pentacosyl-1,3-benzenediol | 0.932771 | 0.192743 |
| Var_343 | Hypogeic acid | 0.931581 | 0.523846 |
| Var_273 | Doxapram | 0.930969 | 0.282529 |
| Var_264 | Dimethyl sulfone | 0.929309 | 0.316337 |
| Var_39 | 24,25-Dihydroxyvitamin D | 0.929055 | 0.373076 |
| Var_11 | 11-Oxo-androsterone glucuronide | 0.928908 | 0.240626 |
| Var_652 | Trifluoroacetic acid | 0.928114 | 0.106474 |
| Var_594 | Sorbitol-6-phosphate | 0.927448 | 0.155551 |
| Var_132 | Amcinonide | 0.925758 | 0.67155 |
| Var_267 | Docosahexaenoic acid | 0.923904 | 0.414519 |
| Var_386 | L-Serine | 0.923699 | 0.191029 |
| Var_345 | Hypoxanthine | 0.923511 | 0.905068 |
| Var_468 | noroxymorphone | 0.922855 | 0.282881 |
| Var_139 | Aprindine | 0.922794 | 0.304107 |
| Var_221 | Dehydroepiandrosterone sulfate | 0.921584 | 0.502321 |
| Var_315 | Gallamine Triethiodide | 0.921356 | 0.818492 |
| Var_313 | Fospropofol | 0.92018 | 0.209217 |
| Var_247 | DG_20:0_16:0_0:0_ | 0.92004 | 0.106184 |
| Var_601 | Succimer | 0.916442 | 0.100609 |
| Var_128 | alpha-Tocopherol | 0.915864 | 0.453892 |
| Var_363 | L-Cysteine | 0.915165 | 0.161973 |
| Var_567 | Resolvin D1 | 0.914978 | 0.505103 |
| Var_319 | Gentamicin | 0.913826 | 0.498951 |
| Var_604 | Sulfanilamide | 0.913369 | 0.098227 |
| Var_429 | Misoprostol | 0.913267 | 0.242519 |
| Var_541 | Prednisone | 0.911951 | 0.214131 |
| Var_87 | 5-Androstenetriol | 0.911599 | 0.106976 |
| Var_653 | Trihydroxycoprostanoic acid | 0.911499 | 0.73879 |
| Var_452 | Nicotinic acid | 0.91068 | 0.208179 |
| Var_633 | Thromboxane A2 | 0.909708 | 0.296957 |
| Var_334 | Heptabarbital | 0.908147 | 0.239357 |
| Var_620 | Terconazole | 0.907835 | 0.337002 |
| Var_527 | Phosphoserine | 0.905679 | 0.315744 |
| Var_372 | Linoleic acid | 0.90477 | 0.554705 |
| Var_433 | Myricetin | 0.904642 | 0.093118 |
| Var_205 | Cladribine | 0.904463 | 0.403351 |
| Var_623 | Tetrahydrodeoxycorticosterone | 0.903958 | 0.352341 |
| Var_473 | Oleic acid | 0.902038 | 0.318659 |
| Var_608 | Sulfisoxazole | 0.900569 | 0.300938 |
| Var_141 | Arformoterol | 0.899889 | 0.120884 |
| Var_472 | Oleanolic acid | 0.898298 | 0.510084 |
| Var_410 | Mesoridazine | 0.897867 | 0.113757 |
| Var_327 | Glycerol | 0.897641 | 0.10701 |
| Var_536 | Pipobroman | 0.896806 | 0.342392 |
| Var_6 | 1_-hydroxytriazolam | 0.894833 | 0.517191 |
| Var_8 | 10Z-Heptadecenoic acid | 0.894256 | 0.35948 |
| Var_158 | Bicalutamide | 0.891395 | 0.181046 |
| Var_138 | Anileridine | 0.889283 | 0.133392 |
| Var_48 | 2-Pyrrolidinone | 0.889215 | 0.059875 |
| Var_602 | Sufentanil | 0.888816 | 0.102855 |
| Var_587 | SM_d16:1_24:1_15Z_ | 0.885966 | 0.156533 |
| Var_127 | alpha-Ketoisovaleric acid | 0.885249 | 0.20542 |
| Var_492 | PC_14:0_18:2_9Z,12Z_ | 0.884946 | 0.162534 |
| Var_436 | Nabumetone | 0.884264 | 0.399679 |
| Var_179 | Cefazolin | 0.884197 | 0.120043 |
| Var_495 | PC_20:1_11Z_14:1_9Z_ | 0.884096 | 0.219662 |
| Var_236 | DG_16:0_22:4_7Z,10Z,13Z,16Z_0:0_ | 0.882675 | 0.759853 |
| Var_193 | Chlorpromazine | 0.881804 | 0.094546 |
| Var_586 | Sitaxentan | 0.881044 | 0.219578 |
| Var_434 | Myristic acid | 0.880642 | 0.408809 |
| Var_317 | gamma-Glutamylcysteine | 0.880264 | 0.108318 |
| Var_286 | Enterodiol | 0.880249 | 0.468309 |
| Var_479 | Orciprenaline | 0.88003 | 0.464132 |
| Var_67 | 3-Methylcrotonylglycine | 0.879613 | 0.348315 |
| Var_373 | Liothyronine | 0.878999 | 0.153644 |
| Var_543 | Procaine | 0.878447 | 0.148725 |
| Var_150 | Azithromycin | 0.87745 | 0.12729 |
| Var_585 | Sevoflurane | 0.877184 | 0.199436 |
| Var_95 | 5-Hydroxymethyl tolterodine | 0.876026 | 0.112649 |
| Var_60 | 3-hydroxyropivacaine | 0.875924 | 0.582061 |
| Var_3 | _2b,3b_-Dihydroxy-30-nor-12,20_29_-oleanadiene-28-glucopyranosyloxy-23-oic acid 3-glucuronide | 0.874153 | 0.153398 |
| Var_569 | Retinoyl b-glucuronide | 0.873358 | 0.723617 |
| Var_402 | LysoPC_P-16:0_ | 0.873071 | 0.259144 |
| Var_534 | Pioglitazone | 0.872436 | 0.260888 |
| Var_146 | Astaxanthin | 0.871777 | 0.182982 |
| Var_298 | Etomidate | 0.871513 | 0.149822 |
| Var_328 | Glycerol 3-phosphate | 0.871192 | 0.091075 |
| Var_120 | Alcoifosfamide | 0.870756 | 0.221344 |
| Var_91 | 5-Heneicosyl-1,3-benzenediol | 0.868984 | 0.175535 |
| Var_424 | Metrizamide | 0.86847 | 0.068237 |
| Var_330 | Glycocholic acid | 0.867677 | 0.652953 |
| Var_591 | SM_d18:0_24:0_ | 0.867533 | 0.125737 |
| Var_593 | Sorafenib beta-D-Glucuronide | 0.86739 | 0.423048 |
| Var_420 | Methylscopolamine | 0.867173 | 0.077822 |
| Var_103 | 6-Thioguanosine monophosphate | 0.867001 | 0.165146 |
| Var_165 | Brimonidine | 0.86676 | 0.434169 |
| Var_598 | Stanozolol | 0.866364 | 0.15865 |
| Var_174 | Carboprost Tromethamine | 0.864702 | 0.13826 |
| Var_18 | 13-Demethyl tacrolimus | 0.864519 | 0.155537 |
| Var_256 | Diflunisal | 0.864484 | 0.104381 |
| Var_455 | Nitisinone | 0.863253 | 0.352153 |
| Var_542 | Pristanic acid | 0.862713 | 0.622696 |
| Var_124 | all-trans-5,6-Epoxyretinoic acid | 0.862563 | 0.662967 |
| Var_672 | Verapamil | 0.862185 | 0.093105 |
| Var_105 | 7-Methylxanthine | 0.861886 | 0.431291 |
| Var_663 | Uracil | 0.860851 | 0.247935 |
| Var_54 | 3-Hydroxybutyric acid | 0.860247 | 0.297585 |
| Var_500 | PC_P-18:1_9Z_22:2_13Z,16Z_ | 0.859993 | 0.115609 |
| Var_418 | Methylergonovine | 0.859848 | 0.675474 |
| Var_636 | Ticagrelor | 0.859196 | 0.135111 |
| Var_297 | Etiocholanediol | 0.858179 | 0.102082 |
| Var_678 | Zonisamide | 0.856835 | 0.210896 |
| Var_304 | Flavoxate | 0.855367 | 0.144324 |
| Var_53 | 3beta-Hydroxypregn-5-en-20-one sulfate | 0.854579 | 0.341925 |
| Var_219 | Decanoylcarnitine | 0.853326 | 0.218008 |
| Var_287 | Epipregnanolone | 0.853307 | 0.099303 |
| Var_437 | N-Acetyl-L-aspartic acid | 0.851796 | 0.071826 |
| Var_517 | PG_18:3_6Z,9Z,12Z_22:6_4Z,7Z,10Z,13Z,16Z,19Z_ | 0.851398 | 0.151741 |
| Var_466 | Norgestimate | 0.850338 | 0.080492 |
| Var_163 | Bopindolol | 0.850151 | 0.123318 |
| Var_338 | Homovanillic acid | 0.849792 | 0.196788 |
| Var_662 | Undecanedioic acid | 0.849399 | 0.837597 |
| Var_151 | Bambuterol | 0.848924 | 0.093128 |
| Var_349 | Imidazolone | 0.848389 | 0.222252 |
| Var_478 | O-Phosphothreonine | 0.847346 | 0.103107 |
| Var_238 | DG_16:1_9Z_20:3_8Z,11Z,14Z_0:0_ | 0.847223 | 0.517867 |
| Var_167 | Brompheniramine | 0.846326 | 0.121209 |
| Var_590 | SM_d18:0_14:1_9Z_OH_ | 0.845271 | 0.126598 |
| Var_514 | Perfluorooctane sulfonamide | 0.845246 | 0.075479 |
| Var_673 | Vilazodone | 0.843631 | 0.164763 |
| Var_573 | Rifampin | 0.843434 | 0.189389 |
| Var_89 | 5a-Tetrahydrocorticosterone | 0.840739 | 0.174053 |
| Var_359 | L-2,4-diaminobutyric acid | 0.840707 | 0.227948 |
| Var_480 | Oseltamivir | 0.838637 | 0.63553 |
| Var_15 | 12-HEPE | 0.838474 | 0.240544 |
| Var_624 | Tetrahydrofuran | 0.838188 | 0.290115 |
| Var_412 | meta-O-Dealkylated flecainide | 0.838023 | 0.282967 |
| Var_178 | CE_22:0_ | 0.836981 | 0.07946 |
| Var_605 | Sulfaphenazole | 0.836545 | 0.357388 |
| Var_648 | Trichloroethanol glucuronide | 0.836175 | 0.069529 |
| Var_430 | Mitomycin | 0.836004 | 0.216175 |
| Var_401 | lysoPC_28:1_5Z_ | 0.83557 | 0.115253 |
| Var_463 | Norbuprenorphine | 0.834516 | 0.087991 |
| Var_75 | 4-Heptanone | 0.83423 | 0.056859 |
| Var_230 | DG_14:0_16:1_9Z_0:0_ | 0.833868 | 0.347461 |
| Var_504 | P-Dichlorobenzene | 0.832946 | 0.179555 |
| Var_229 | Dextrothyroxine | 0.832524 | 0.100098 |
| Var_320 | Gentian Violet | 0.832372 | 0.275076 |
| Var_655 | Trimethobenzamide | 0.831889 | 0.145893 |
| Var_411 | Metabolite M6 | 0.831441 | 0.103914 |
| Var_51 | 3,4-Dihydroxyphenylglycol | 0.831362 | 0.273626 |
| Var_80 | 4-Hydroxyproline | 0.83081 | 0.150825 |
| Var_100 | 5-Tricosyl-1,3-benzenediol | 0.830136 | 0.232014 |
| Var_186 | Ceramide _d18:1_25:0_ | 0.829872 | 0.109337 |
| Var_625 | TG_16:0_14:0_18:2_9Z,12Z_ | 0.828699 | 0.13442 |
| Var_110 | Acebutolol | 0.828486 | 0.303799 |
| Var_32 | 2,3-Butanediol | 0.828116 | 0.150688 |
| Var_494 | PC_18:3_6Z,9Z,12Z_16:0_ | 0.827569 | 0.145791 |
| Var_175 | Carphenazine | 0.827348 | 0.087894 |
| Var_61 | 3-Hydroxytamoxifen _Droloxifene_ | 0.825528 | 0.16437 |
| Var_659 | Tyramine glucuronide | 0.823474 | 0.20651 |
| Var_162 | Bismuth Subsalicylate | 0.823006 | 0.131222 |
| Var_25 | 17-beta-Estradiol glucuronide | 0.822773 | 0.79782 |
| Var_370 | Lidocaine | 0.82115 | 0.096913 |
| Var_506 | Pefloxacin | 0.820898 | 0.440398 |
| Var_278 | Drotaverine | 0.819455 | 0.174103 |
| Var_71 | 4-_Methylnitrosamino_-1-_3-pyridyl_-1-butanol glucuronide | 0.819061 | 0.12298 |
| Var_574 | Rimexolone | 0.818657 | 0.055191 |
| Var_634 | Thromboxane B2 | 0.818448 | 0.195304 |
| Var_497 | PC_o-16:1_9Z_14:1_9Z_ | 0.818007 | 0.136775 |
| Var_293 | Escitalopram | 0.817442 | 0.177509 |
| Var_376 | Lithocholic acid glycine conjugate | 0.817346 | 0.217579 |
| Var_157 | Bexarotene | 0.813445 | 0.106495 |
| Var_557 | Quinidine | 0.811045 | 0.110276 |
| Var_528 | Phthalic acid | 0.809533 | 0.11394 |
| Var_38 | 20-HETE ethanolamide | 0.808928 | 0.305402 |
| Var_97 | 5-O-Desmethyldonepezil | 0.808762 | 0.323021 |
| Var_295 | Ethanol | 0.808679 | 0.155279 |
| Var_535 | Pipecuronium | 0.808643 | 0.14963 |
| Var_571 | Retinyl ester | 0.808617 | 0.121689 |
| Var_199 | Cinacalcet | 0.808107 | 0.108701 |
| Var_262 | Dihydrothymine | 0.807714 | 0.203349 |
| Var_200 | Cinitapride | 0.807453 | 0.306412 |
| Var_441 | Nalbuphine | 0.807412 | 0.371033 |
| Var_240 | DG_18:1_11Z_16:1_9Z_0:0_ | 0.807149 | 0.434971 |
| Var_234 | DG_14:1_9Z_22:0_0:0_ | 0.807129 | 0.174591 |
| Var_533 | Pindolol | 0.806596 | 0.191608 |
| Var_408 | Mechlorethamine | 0.80645 | 0.13583 |
| Var_637 | Tiludronate | 0.805078 | 0.140557 |
| Var_460 | Nitroglycerin | 0.804919 | 0.0524 |
| Var_496 | PC_24:1_15Z_P-18:1_11Z_ | 0.800683 | 0.050516 |
| Var_268 | Docosapentaenoic acid _22n-6_ | 0.799672 | 0.257605 |
| Var_346 | Ibutilide | 0.798472 | 0.069323 |
| Var_160 | Biopterin | 0.796716 | 0.097324 |
| Var_166 | Bromodichloromethane | 0.795092 | 0.431643 |
| Var_428 | Milrinone | 0.793377 | 0.036107 |
| Var_589 | SM_d17:1_24:1_15Z_ | 0.791854 | 0.084078 |
| Var_609 | Sulfite | 0.788593 | 0.71276 |
| Var_194 | Cholestane-3,7,12,25-tetrol-3-glucuronide | 0.788251 | 0.16181 |
| Var_397 | LysoPC_22:5_4Z,7Z,10Z,13Z,16Z_ | 0.784758 | 0.433711 |
| Var_595 | Sphinganine 1-phosphate | 0.784202 | 0.290072 |
| Var_344 | Hypotaurine | 0.78285 | 0.18939 |
| Var_332 | Guanfacine | 0.782128 | 0.12491 |
| Var_81 | 4-oxo-Retinoic acid | 0.782076 | 0.131236 |
| Var_393 | LysoPC_20:5_5Z,8Z,11Z,14Z,17Z_ | 0.779315 | 0.659475 |
| Var_442 | Nalidixic Acid | 0.779081 | 0.144568 |
| Var_611 | Sunitinib | 0.778678 | 0.336381 |
| Var_183 | Cer_d18:1_14:0_ | 0.777913 | 0.825348 |
| Var_499 | PC_P-18:0_24:0_ | 0.776287 | 0.116544 |
| Var_189 | Chloramphenicol | 0.776122 | 0.198183 |
| Var_134 | Aminoadipic acid | 0.775011 | 0.193934 |
| Var_153 | Bepridil | 0.774351 | 0.396303 |
| Var_638 | Tinidazole | 0.77414 | 0.287516 |
| Var_62 | 3-Hydroxytetradecanedioic acid | 0.773948 | 0.345924 |
| Var_303 | Fexofenadine | 0.773086 | 0.177667 |
| Var_33 | 2,3-diene-Valproic acid-CoA | 0.771163 | 0.564718 |
| Var_46 | 2-hydroxymethylolanzapine | 0.771116 | 0.302123 |
| Var_498 | PC_o-22:0_20:4_8Z,11Z,14Z,17Z_ | 0.7706 | 0.15206 |
| Var_488 | Palmitic acid | 0.769529 | 0.17335 |
| Var_631 | Thiosulfate | 0.767082 | 0.077042 |
| Var_70 | 3-Sulfinoalanine | 0.765163 | 0.44824 |
| Var_362 | Lanthionine ketimine | 0.763366 | 0.287074 |
| Var_382 | Lornoxicam | 0.763184 | 0.077918 |
| Var_448 | N-Despyridinyl rosiglitazone | 0.762835 | 0.731576 |
| Var_172 | Carbamazepine-O-quinone | 0.762753 | 0.300458 |
| Var_211 | Cortexolone | 0.760929 | 0.387668 |
| Var_284 | Eletriptan N-oxide | 0.760545 | 0.357833 |
| Var_588 | SM_d17:1_24:0_ | 0.759124 | 0.175882 |
| Var_414 | Metharbital | 0.757487 | 0.571088 |
| Var_214 | Cyclandelate | 0.755801 | 0.204555 |
| Var_272 | Dopamine 3-O-sulfate | 0.755495 | 0.149423 |
| Var_447 | N-Desmethyldiltiazem | 0.755438 | 0.168075 |
| Var_299 | Etonogestrel | 0.755116 | 0.303969 |
| Var_521 | Phenprocoumon | 0.755012 | 0.75486 |
| Var_449 | Neurosporene | 0.754717 | 0.298885 |
| Var_649 | Tricin 7-_feruloyl-_-_2_-glucuronyl-_1-_2_-glucuronide_ | 0.753257 | 0.07786 |
| Var_231 | DG_14:0_18:0_0:0_ | 0.752987 | 0.384137 |
| Var_289 | Ergothioneine | 0.752644 | 0.365678 |
| Var_613 | Tamoxifen | 0.751143 | 0.115509 |
| Var_144 | Ascorbic acid-2-sulfate | 0.750945 | 0.321811 |
| Var_82 | 5,6-DHET | 0.750421 | 0.137846 |
| Var_188 | Chenodeoxycholic acid glycine conjugate | 0.749991 | 0.786306 |
| Var_562 | Reboxetine | 0.747802 | 0.217684 |
| Var_281 | Dyphylline | 0.7478 | 0.313981 |
| Var_619 | Tenoxicam | 0.746943 | 0.377174 |
| Var_597 | SR 49498 | 0.746755 | 0.424744 |
| Var_531 | PI_18:1_11Z_20:4_5Z,8Z,11Z,14Z_ | 0.744638 | 0.039841 |
| Var_568 | Retinal | 0.744274 | 0.394155 |
| Var_525 | Phloretin | 0.74424 | 0.306833 |
| Var_374 | Lisuride | 0.744197 | 0.159405 |
| Var_530 | PI_16:0_20:2_11Z,14Z_ | 0.743037 | 0.094362 |
| Var_26 | 17-Beta-Estradiol-3,17-beta-sulfate | 0.742033 | 0.538632 |
| Var_113 | Acetic acid | 0.740897 | 0.561339 |
| Var_367 | Levocabastine | 0.7407 | 0.126 |
| Var_197 | Cilastatin | 0.740241 | 0.375729 |
| Var_671 | Ursodeoxycholic acid 3-sulfate | 0.737688 | 0.466763 |
| Var_239 | DG_18:0_18:2_9Z,12Z_0:0_ | 0.737617 | 0.18461 |
| Var_255 | Dicyclomine | 0.737505 | 0.360202 |
| Var_192 | Chloroform | 0.737005 | 0.14573 |
| Var_24 | 17-beta-estradiol 3-sulfate-17-_beta-D-glucuronide_ | 0.733694 | 0.086455 |
| Var_109 | 9,10-Epoxyoctadecenoic acid | 0.733377 | 0.644251 |
| Var_79 | 4-Hydroxycyclophosphamide | 0.733227 | 0.275336 |
| Var_245 | DG_18:2_9Z,12Z_14:1_9Z_0:0_ | 0.730664 | 0.20077 |
| Var_406 | Malondialdehyde | 0.730608 | 0.416463 |
| Var_190 | Chlorhexidine | 0.729508 | 0.203504 |
| Var_484 | Oxybutynin | 0.726726 | 0.289427 |
| Var_651 | Tridihexethyl | 0.726077 | 0.135175 |
| Var_260 | Dihydrodaidzein | 0.725006 | 0.189433 |
| Var_92 | 5-Hydroxyfluvastatin | 0.723477 | 0.873168 |
| Var_470 | O-Desmethyltramadol glucuronide | 0.722723 | 0.43027 |
| Var_628 | TG_18:2_9Z,12Z_18:1_9Z_18:3_6Z,9Z,12Z_ | 0.72124 | 0.127681 |
| Var_391 | LysoPC_16:1_9Z_0:0_ | 0.718415 | 0.218988 |
| Var_668 | Uridine 5_-monophosphate | 0.716545 | 0.140952 |
| Var_85 | 5-_3_-Hydroxyphenyl_-gamma-valerolactone-3_-O-glucuronide | 0.71515 | 0.396312 |
| Var_350 | Indinavir | 0.711081 | 0.175004 |
| Var_47 | 2-Phosphoglyceric acid | 0.707596 | 0.190291 |
| Var_235 | DG_14:1_9Z_22:4_7Z,10Z,13Z,16Z_0:0_ | 0.70674 | 0.497573 |
| Var_321 | Glisoxepide | 0.705128 | 0.360363 |
| Var_635 | Thromboxane B3 | 0.702925 | 0.07943 |
| Var_22 | 15_S_-Hydroxyeicosatrienoic acid | 0.701519 | 0.568794 |
| Var_123 | Allopurinol riboside | 0.70109 | 1.09226 |
| Var_337 | Homocysteine thiolactone | 0.697011 | 0.440724 |
| Var_276 | D-Ribose 5-phosphate | 0.696188 | 0.238217 |
| Var_291 | Erucic acid | 0.692151 | 0.580958 |
| Var_288 | Eplerenone | 0.690792 | 0.327299 |
| Var_305 | Flumethasone Pivalate | 0.689487 | 0.207737 |
| Var_83 | 5,6-Dihydro-5-fluorouracil | 0.687838 | 0.160161 |
| Var_560 | Ranitidine | 0.68609 | 0.238377 |
| Var_96 | 5-Methylcytidine | 0.685231 | 0.132208 |
| Var_43 | 2-Hexenoylcarnitine | 0.684185 | 0.249818 |
| Var_576 | Ritonavir | 0.682564 | 0.226957 |
| Var_454 | Nimodipine | 0.681691 | 0.490283 |
| Var_159 | Bilirubin glucuronide | 0.680414 | 0.165445 |
| Var_515 | Perfluorooctanesulfonic acid | 0.678724 | 0.438657 |
| Var_227 | Dexamethasone | 0.67818 | 0.106849 |
| Var_208 | Coenzyme Q9 | 0.677388 | 0.174475 |
| Var_440 | NAD | 0.676913 | 0.110551 |
| Var_90 | 5a-Tetrahydrocortisol | 0.676545 | 0.323198 |
| Var_627 | TG_18:1_9Z_16:0_20:2_11Z,14Z_ | 0.676055 | 0.094833 |
| Var_228 | Dextrorphan O-glucuronide | 0.674974 | 0.126231 |
| Var_510 | Pentazocine | 0.671097 | 0.272111 |
| Var_329 | Glycochenodeoxycholate-3-sulfate | 0.670469 | 0.193325 |
| Var_491 | PC_14:0_18:1_9Z_ | 0.666382 | 0.246553 |
| Var_202 | Cisplatin | 0.665075 | 0.155072 |
| Var_195 | Cholesterol sulfate | 0.663706 | 0.141302 |
| Var_660 | Ubiquinone-1 | 0.662483 | 0.093231 |
| Var_353 | Irinotecan | 0.661537 | 0.396618 |
| Var_464 | Norcodeine | 0.655851 | 0.468979 |
| Var_381 | Lorazepam | 0.654708 | 0.688581 |
| Var_296 | Etidronic acid | 0.654454 | 0.340801 |
| Var_371 | Lindane | 0.6544 | 0.393698 |
| Var_621 | Testolactone | 0.650728 | 0.262825 |
| Var_270 | Dodecanoic acid | 0.648713 | 0.5824 |
| Var_253 | Dichlorphenamide | 0.64755 | 0.16218 |
| Var_493 | PC_14:0_P-16:0_ | 0.646829 | 0.329845 |
| Var_461 | N-Monodesmethyl-rizatriptan | 0.646121 | 0.356363 |
| Var_116 | Aciclovir | 0.644349 | 0.592034 |
| Var_394 | LysoPC_22:1_13Z_ | 0.640514 | 0.203265 |
| Var_462 | NNAL-N-glucuronide | 0.639665 | 0.278526 |
| Var_579 | Roxatidine acetate | 0.638872 | 0.271092 |
| Var_553 | Quercetin 3,4_-diglucoside | 0.638531 | 0.291911 |
| Var_126 | Almitrine | 0.636326 | 0.663422 |
| Var_42 | 2-ene-Valproic acid CoA | 0.636288 | 0.062888 |
| Var_476 | OP-1118 | 0.634233 | 0.131489 |
| Var_69 | 3-O-Methylrosmarinic acid | 0.632226 | 0.718639 |
| Var_544 | Promethazine | 0.63179 | 0.313157 |
| Var_36 | 2,5-Furandicarboxylic acid | 0.630461 | 0.419817 |
| Var_244 | DG_18:1_9Z_18:2_9Z,12Z_0:0_ | 0.629173 | 0.410194 |
| Var_259 | Dihydrocaffeic acid 3-sulfate | 0.62796 | 0.128499 |
| Var_290 | Erlotinib | 0.625856 | 0.127227 |
| Var_342 | Hydroxykynurenine | 0.62569 | 0.12611 |
| Var_232 | DG_14:0_20:3_8Z,11Z,14Z_0:0_ | 0.625208 | 0.143216 |
| Var_112 | Acetamide | 0.624758 | 0.126671 |
| Var_508 | Pentadecanoic acid | 0.62037 | 0.500209 |
| Var_249 | D-Glyceraldehyde 3-phosphate | 0.617581 | 0.035648 |
| Var_489 | Pamidronate | 0.616378 | 0.137356 |
| Var_365 | Leukotriene D4 | 0.612087 | 0.420957 |
| Var_258 | Dihydrobiopterin | 0.611871 | 0.394909 |
| Var_31 | 1-Methylinosine | 0.611244 | 0.679615 |
| Var_130 | Alprazolam | 0.611039 | 0.521851 |
| Var_596 | Spironolactone | 0.607848 | 0.475328 |
| Var_518 | PG_18:3_9Z,12Z,15Z_20:3_5Z,8Z,11Z_ | 0.607388 | 0.495097 |
| Var_99 | 5-Tetradecenoic acid | 0.606059 | 1.00902 |
| Var_311 | fluvoxamino acid | 0.605956 | 0.169029 |
| Var_656 | Tripelennamine | 0.604924 | 0.12904 |
| Var_471 | Oleamide | 0.6015 | 0.466735 |
| Var_196 | Cholic acid glucuronide | 0.600413 | 0.197912 |
| Var_2 | _23S_-23,25-dihdroxy-24-oxovitamine D3 23-_beta-glucuronide_ | 0.597142 | 0.243722 |
| Var_49 | 3,4-Dihydroxymandelic acid | 0.590961 | 0.323833 |
| Var_191 | Chlormezanone | 0.587752 | 0.281586 |
| Var_432 | Morphine-3-glucuronide | 0.58575 | 0.171629 |
| Var_331 | Gossypetin 8-glucuronide 3-sulfate | 0.5852 | 0.083073 |
| Var_538 | Pivmecillinam | 0.572208 | 0.219946 |
| Var_645 | Trazodone | 0.569021 | 0.440823 |
| Var_65 | 3-Methoxymorphinan | 0.568357 | 0.534439 |
| Var_511 | Pentostatin | 0.567768 | 0.305948 |
| Var_610 | Sulfolithocholylglycine | 0.563244 | 0.257294 |
| Var_185 | Ceramide _d18:1_22:0_ | 0.551806 | 0.2509 |
| Var_563 | Reduced haloperidol | 0.5462 | 0.266772 |
| Var_547 | PS_18:1_9Z_22:6_4Z,7Z,10Z,13Z,16Z,19Z_ | 0.541306 | 0.180713 |
| Var_657 | Tropicamide | 0.539576 | 0.376006 |
| Var_294 | Estriol-3-glucuronide | 0.538609 | 0.178819 |
| Var_423 | Metocurine | 0.538435 | 0.516307 |
| Var_626 | TG_16:0_18:1_9Z_18:2_9Z,12Z_ | 0.533488 | 0.312204 |
| Var_77 | 4-Hydroxy-5-_phenyl_-valeric acid-O-glucuronide | 0.531318 | 0.137057 |
| Var_578 | Rosuvastatin | 0.528412 | 0.242001 |
| Var_302 | Feruloyl C1-glucuronide | 0.527951 | 0.254387 |
| Var_152 | Benzonatate | 0.520008 | 0.236029 |
| Var_64 | 3-keto Fusidic acid | 0.512584 | 0.164169 |
| Var_559 | Ramipril Diketopiperazine | 0.507863 | 0.083826 |
| Var_583 | Sertindole | 0.502353 | 0.105629 |
| Var_532 | Pi-Methylimidazoleacetic acid | 0.4929 | 0.149311 |
| Var_182 | Cer_d18:0_14:0_ | 0.489319 | 0.479028 |
| Var_524 | Phenytoin quinone | 0.482605 | 0.272809 |
| Var_677 | Zafirlukast metabolite M5 | 0.479778 | 0.295696 |
| Var_617 | Tazobactam | 0.478274 | 0.225061 |
| Var_251 | Diazepam | 0.475386 | 0.365524 |
| Var_395 | LysoPC_22:2_13Z,16Z_ | 0.46837 | 0.848881 |
| Var_502 | p-Cresol glucuronide | 0.466537 | 0.400334 |
| Var_453 | Niflumic Acid | 0.461531 | 0.234296 |
| Var_581 | S-Adenosylhomocysteine | 0.438391 | 0.504906 |
| Var_669 | Uroporphyrinogen III | 0.425187 | 0.235801 |
| Var_555 | Quercetin-3_-glucuronide | 0.422101 | 0.190297 |
| Var_674 | Warfarin | 0.405379 | 0.260652 |
| Var_554 | Quercetin 3_-sulfate | 0.376057 | 0.229858 |
| Var_564 | Remoxipride | 0.370099 | 0.480917 |
| Var_84 | 5-_3_,4_-Dihydroxyphenyl_-gamma-valerolactone-4_-O-methyl-3_-O-glucuronide | 0.362487 | 0.405423 |
| Var_356 | Isorhamnetin | 0.344329 | 0.401733 |
| Var_643 | trans-Nonachlor | 0.310928 | 0.199294 |
| Var_400 | lysoPC_26:0_ | 0.29882 | 0.320513 |
| Var_600 | Streptomycin | 0.29338 | 0.392848 |
| Var_558 | rac-5,6-Epoxy-retinoyl-beta-D-glucuronide | 0.28127 | 0.410904 |
| Var_501 | p-Chlorobenzene sulfonyl urea | 0.270318 | 0.15045 |
| Var_17 | 13,14-Dihydro-15-keto PGF2a | 0.258846 | 0.566794 |
| Var_210 | Coproporphyrinogen III | 0.181503 | 0.19484 |
| Var_34 | 2,3-Diphosphoglyceric acid | 0.162511 | 0.476324 |

**Supplementary Table S5**

|  | Total | Expected | Hits | p value | Impact |
| --- | --- | --- | --- | --- | --- |
| Phenylalanine metabolism | 10 | 0.26452 | 3 | 0.001814 | 0.35714 |
| Phenylalanine, tyrosine and tryptophan biosynthesis | 4 | 0.10581 | 2 | 0.003962 | 1 |
| Arginine biosynthesis | 14 | 0.37032 | 2 | 0.050822 | 0.11675 |
| Histidine metabolism | 16 | 0.42323 | 2 | 0.064834 | 0.22131 |
| Citrate cycle (TCA cycle) | 20 | 0.52903 | 2 | 0.096106 | 0.14743 |
| beta-Alanine metabolism | 21 | 0.55548 | 2 | 0.10449 | 0.39925 |
| D-Glutamine and D-glutamate metabolism | 6 | 0.15871 | 1 | 0.1488 | 0.5 |
| Alanine, aspartate and glutamate metabolism | 28 | 0.74065 | 2 | 0.16784 | 0.19712 |
